# In Operando X-ray Spectroscopic and DFT Studies Revealing Improved H_2_ Evolution by the Synergistic Ni–Co Electron Effect in the Alkaline Condition

**DOI:** 10.1021/acsami.4c02613

**Published:** 2024-05-20

**Authors:** Lian-Ming Lyu, Han-Jung Li, Ren-Shiang Tsai, Ching-Feng Chen, Yu-Chung Chang, Yu-Chun Chuang, Cheng-Shiuan Li, Jeng-Lung Chen, Te-Wei Chiu, Chun-Hong Kuo

**Affiliations:** † Department of Applied Chemistry, National Yang Ming Chiao Tung University, Hsinchu 300093, Taiwan; ‡ Center for Emergent Functional Matter Science, National Yang Ming Chiao Tung University, Hsinchu 300093, Taiwan; § Department of Materials and Mineral Resources Engineering, Institute of Materials Science and Engineering, National Taipei University of Technology, Taipei 106344, Taiwan; ∥ National Synchrotron Radiation Research Center, Hsinchu 300092, Taiwan; ⊥ Green Energy and Environment Research Laboratories, Industrial Technology Research Institute, Hsinchu 310401, Taiwan

**Keywords:** HER, catalysis, operando, X-ray, DFT

## Abstract

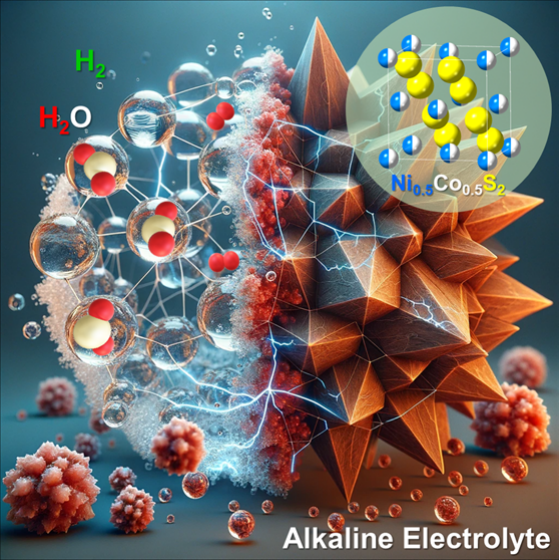

The different electrolyte conditions, e.g., pH value, for driving efficient HER and OER are one of the major issues hindering the aim for electrocatalytic water splitting in a high efficiency. In this regard, seeking durable and active HER electrocatalysts to align the alkaline conditions of the OER is a promising solution. However, the success in this strategy will depend on a fundamental understanding about the HER mechanism at the atomic scale. In this work, we have provided thorough understanding for the electrochemical HER mechanisms in KOH over Ni- and Co-based hollow pyrite microspheres by in operando X-ray spectroscopies and DFT calculations, including NiS_2_, CoS_2_, and Ni_0.5_Co_0.5_S_2_. We discovered that the Ni sites in hollow NiS_2_ microspheres were very stable and inert, while the Co sites in hollow CoS_2_ microspheres underwent reduction and generated Co metallic crystal domains under HER. The generation of Co metallic sites would further deactivate H_2_ evolution due to the large hydrogen desorption free energy (−1.73 eV). In contrast, the neighboring Ni and Co sites in hollow Ni_0.5_Co_0.5_S_2_ microspheres exhibited the electronic interaction to elevate the reactivity of Ni and facilitate the stability of Co without structure or surface degradation. The energy barrier in H_2_O adsorption/dissociation was only 0.73 eV, followed by 0.06 eV for hydrogen desorption over the Ni_0.5_Co_0.5_S_2_ surface, revealing Ni_0.5_Co_0.5_S_2_ as a HER electrocatalyst with higher durability and activity than NiS_2_ and CoS_2_ in the alkaline medium due to the synergy of neighboring Ni and Co sites. We believe that the findings in our work offer a guidance toward future catalyst design.

## Introduction

Hydrogen gas (H_2_) is the cleanest source for energy generation without carbon emission, hence urging the technique development of the hydrogen evolution reaction (HER). H_2_ evolution from water electrolysis is a traditional but direct way to produce clean energy for meeting global energy crisis.^1−3^ By referring to the prediction in Yan’s work, Ni- and Co-based pyrites (NiS_2_ and CoS_2_) can be good candidates for efficient HER in acidic media.^4−10^ Unfortunately, the oxygen evolution reaction (OER) over the Ni- and Co-based pyrite electrocatalysts is usually conducted in the alkaline electrolytes for high efficiency.^11−15^ Such two conflicting conditions unlikely lead to efficient total water splitting. In this regard, seeking durable and active HER electrocatalysts to align the alkaline conditions of the OER can be one of the solutions. However, the success in this strategy will rely on a thorough understanding about the HER mechanism in the alkaline medium. So far, the HER mechanisms over electrocatalysts are mostly explained from the viewpoints of thermodynamics by DFT calculations (the proton adsorption energy), which possibly neglect some critical clues in the real reaction process. Hence, temporal spectroscopy has become an emerging tool for making up the lost pieces in the stories of reaction mechanisms.

The cubic pyrite-phase transition metal dichalcogenides (with the general formula MX_2_, where typically M = Fe, Co, or Ni and X = S or Se) have been considered as efficient HER electrocatalysts since 2014.^16−18^ Inspired by natural hydrogenase enzymes, which contain metal sulfides as the active sites for HER, it is highly desirable to create artificial metal sulfides to mimic the natural HER process.^8^ Within the family of transition metal pyrites, iron disulfide (iron pyrite, FeS_2_), cobalt disulfide (cobalt pyrite, CoS_2_), and nickel disulfide (nickel pyrite, NiS_2_) particularly attract attention for their high abundances and superior activities.

**Figure 1:**
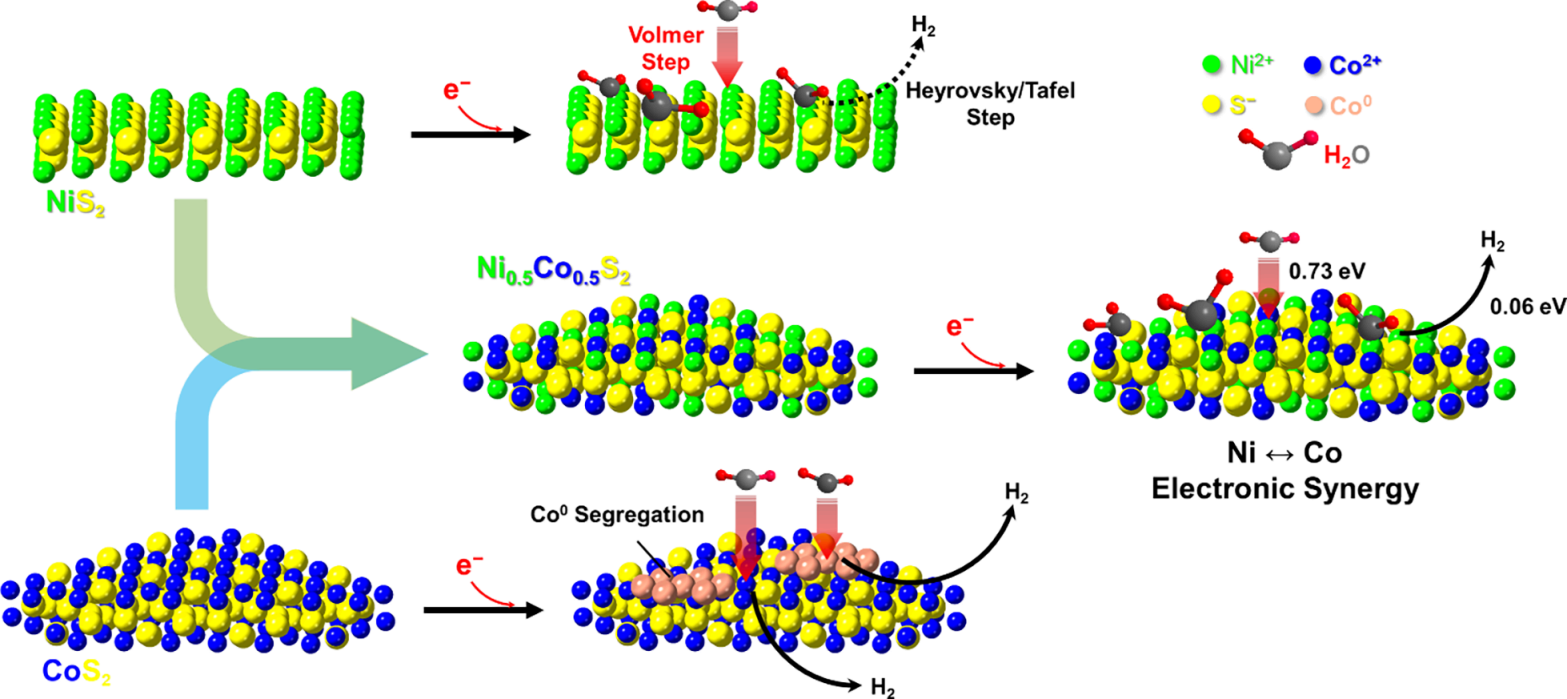
Schematic hypothesis for our findings in the differences of HER mechanisms over the hollow NiS_2_, Ni_0.5_Co_0.5_S_2_, and CoS_2_ microsphere surfaces.

In this work, we examined the HER efficiencies and kinetics of the pyrite NiS_2_, CoS_2_ and Ni_0.5_Co_0.5_S_2_ materials (electrocatalysts) in the alkaline electrolyte (KOH), and we provided insights into their HER mechanisms through cooperative analyses by in operando X-ray spectroscopies and density functional theory (DFT) calculations. Figure 1 is the schematic hypothesis to support our observations on the improved H_2_ evolution by hollow Ni_0.5_Co_0.5_S_2_ microspheres. On the surfaces of hollow NiS_2_ (100 faces in majority) and Ni_0.5_Co_0.5_S_2_ microspheres (111 faces in majority), the structures are stable in KOH under the applied potentials confirmed by in operando XRD and XAS spectroscopies. However, the surfaces of hollow CoS_2_ microspheres (111 faces in the majority) undergo reduction and generate Co metallic crystal domains. From DFT calculations, the energy barrier in the first step (Volmer step) of H_2_O adsorption/dissociation in alkaline HER is 0.73 eV and the free energy of the following hydrogen desorption (Heyrovsky/Tafel step to form H_2_) is only 0.06 eV over the Ni_0.5_Co_0.5_S_2_ surface. The low energy barriers in both steps render the Ni_0.5_Co_0.5_S_2_(111) face more suitable for alkaline HER than NiS_2_ and CoS_2_. In light of the results, we can demonstrate that the H_2_ evolution over the Ni_0.5_Co_0.5_S_2_ surface is truly improved by the synergistic electronic effect of Ni and Co. The electronic effect promoted Ni_0.5_Co_0.5_S_2_ to exhibit better HER kinetics in KOH than those of NiS_2_ and CoS_2_. In addition, it prevented the Ni_0.5_Co_0.5_S_2_ surface from the issue of Co^0^ segregation that would suppress H_2_ evolution.

## Experimental Section

### Chemicals

Nickel nitrate hexahydrate (Ni(NO_3_)_2_·6H_2_O, 98%, Alfa Aesar), cobalt(II) nitrate hexahydrate (Co(NO_3_)_2_·6H_2_O, ≥98%, Sigma-Aldrich), sodium thiosulfate 5-hydrate (Na_2_S_2_O_3_·5H_2_O 99.5%, J.T.Baker), carbon paper (20 × 50 mm^2^/pc, 0.18 mm thick, CeTech), ethanol (EtOH, 95%, ECHO Chemical), potassium hydroxide (KOH, ≥98%, Sigma-Aldrich), sulfuric acid (H_2_SO_4_, 95.0–97.0%, Honeywell), silicone elastomer A (UniRegion BiO-Tech), silicone elastomer B (UniRegion BiO-Tech), argon (Ar, 99.999%, Cing Fong Co), 2-propanol (C_3_H_8_O, >99.8%, Sigma-Aldrich), and Nafion:perfluorinated resin solution (5 wt % in lower aliphatic alcohols and water, containing 15–20% water, Sigma-Aldrich) were used. In this study, all of the chemicals were purchased from chemical vendors and used without purification.

### Synthesis of Ni_0.5_Co_0.5_S_2_, NiS_2_, and CoS_2_ Microspheres

All kinds of microspheres were synthesized by the hydrothermal method. For the synthesis of Ni_0.5_Co_0.5_S_2_ microspheres, a 10 mL aqueous solution containing 0.5 mmol of Ni(NO_3_)_2_·6H_2_O, 0.5 mmol of Co(NO_3_)_2_·6H_2_O, and 2 mmol of Na_2_S_2_O_3_·5H_2_O was prepared under vigorous stirring for 10 min. Next, the mixed solution was transferred to a Teflon-lined stainless-steel autoclave (20 mL), followed by heating at 180 °C in an air-circulating oven for 12 h. After the reaction, the solution was naturally cooled down to room temperature. At last, the powder in the solution was collected by centrifuging at 5000 rpm and washed with deionized water and pure ethanol in turn. The washing steps were repeated twice, and the powder was finally dried in a vacuum oven at 40 °C overnight. To get the microspheres of NiS_2_ and CoS_2_, 1 mmol of Ni(NO_3_)_2_·6H_2_O or Co(NO_3_)_2_·6H_2_O was solely used instead of 0.5 mmol of Ni(NO_3_)_2_·6H_2_O and 0.5 mmol of Co(NO_3_)_2_·6H_2_O to blend with 2 mmol of Na_2_S_2_O_3_·5H_2_O; otherwise, all steps were the same.

### Characterization

Morphologies and surface states of nanocrystals were analyzed by field-emission scanning electron microscopy operated at an accelerated voltage of 10 keV (FE-SEM, ZEISS ULTRA PLUS instrument with an OXFORD EDX detector). Bright-field TEM and HAADF-STEM-EDS images were acquired by spherical-aberration-corrected field-emission transmission electron microscopy (Cs-corrected TEM, JEOL ARM 200F) operated at 200 keV. The elemental ratios of all samples were measured by inductively coupled plasma with mass spectroscopy (NexION 350 ICP-MS, PerkinElmer). The electronic states of elements for all samples were determined by a high-resolution X-ray photoelectron spectrometer (ULVAC-PHI, PHI Quantera II) equipped with a scanning X-ray microprobe (Al anode) as the X-ray source. The in operando X-ray diffraction and absorption experiments were carried out using the Taiwan Photon Source in the National Synchrotron Radiation Research Center (NSRRC), Taiwan. The X-ray diffraction data were acquired at TPS19A using the X-rays with 20 keV (λ = 0.61992 Å) for both regular sample measurements and in operando measurements and a MYTHEN18K detector with Debye–Scherrer geometry. The patterns were converted and refined by the GSAS-II program. The LaB_6_ (SRM 660c) standard was used as the standard for angle calibration. Every pattern was acquired after applying potentials for 10 min. The XAS spectra including X-ray absorption near-edge structure (XANES) and extended X-ray absorption fine structure (EXAFS) were obtained at TPS44A with the q-scan method in the transmission mode.^19^ Standard metal foils (Ni and Co) were employed as references for the energy calibration of incident photons. Each spectrum was collected with Q-Mono oscillating at 1 Hz for 2 min. Data fitting of XANES and EXAFS was conducted with the Demeter package.^20^

### Electrochemical Hydrogen Evolution Reaction

Chosen as the substrate of the working electrode, the carbon paper was hooked to a copper wire. Next, the silicone elastomer (mix ratio A:B = 10:1) was applied to fix the connecting joint, followed by the electrode heated in an air-circulating oven at 120 °C for 5 min to solidify the elastomer. One milligram of electrocatalysts (microspheres) was dispersed in a mixed solution (20 μL of ethanol and 20 μL of 0.5 wt % Nafion solution). The solution was sonicated for at least 20 min to form homogeneous ink. After that, 40 μL of the ink was loaded onto the carbon paper electrode. The amount of loading was about 0.7 ± 0.2 mg/cm^2^.

All of the measurements of water splitting were carried out on a three-electrode potentiostat (CHI760E, CH Instruments) at room temperature (25 °C). A cylinder-shaped plastic cell was utilized to hold the electrolyte and set the electrodes. Electrochemical experiments were conducted in an electrolyte of 1 M KOH (pH = 13.8 ± 0.1). A graphite rod was used as the counter electrode, and a Hg/HgO electrode was used as the reference electrode. The geometric area of the working electrode immersed in the electrolyte was 0.2 × 0.5 cm^2^ used for data normalization. The detailed investigation of hydrogen evolution (HER) and linear sweep voltammogram (LSV) was done across the range from −0.95 to −1.95 V (vs Hg/HgO) for HER at a scan rate of 5 mV/s. All the results were consequently converted to versus the reversible hydrogen electrode (RHE) by the following equation:
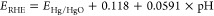
1

The electrochemical double-layer capacitance (*C*_dl_) was evaluated by collecting cyclic voltammograms (CVs) in the potential range with a nonfaradic process at different scan rates (*r*) of 10, 20, 30, 40, 50, 60, 80, and 100 mV/s. The potential windows of ECSA were determined by the open-circuit potential (OCP) of ±0.04 V (vs RHE). Then, the double-layer capacitance (*C*_dl_), which is half of d(Δ*I*)/d(*r*), was estimated by plotting Δ*I*_OCP_ = (*I*_top_ – *I*_bottom_ at OCP) as a function of the scan rate (*r*). The electrochemically active surface areas (ECSAs) were estimated according to the following formula.
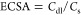
2

*C*_s_ is the specific capacitance of a flat surface with 1 cm^2^ real surface area, which is generally in the range of 0.02 to 0.06 mF/cm^2^. Thus, the averaged value of 0.04 mF/cm^2^ was assumed for the flat electrode.^21,22^ Electrochemical impedance spectroscopy (EIS) was conducted by applying a potential of −0.4 V (vs RHE), an AC frequency of 100 kHz to 1 mHz, and an amplitude of 0.005 V.

### Density Functional Theory Calculations

All calculations were performed with the DFT plane-wave method implemented in the Vienna ab initio simulation package (VASP). The projector-augmented-wave pseudopotentials (400 eV) in conjunction with the PBE density functional were employed.^23−26^ All surfaces were modeled by 4-layer slabs in a (2 × 2) lateral supercell as shown in Figure S8, and the lowest two layers were fixed for all the structural optimizations. To mitigate any undesirable interactions along the *z*-direction, a minimum vacuum of 15 Å was introduced. For the summation in the Brillouin zone, the Monkhorst–Pack mesh k-point was set to (4 × 4× 1) on all surfaces.^27^ The adsorption energy of H (Δ*E*_H*_) was calculated based on the equation as follows:
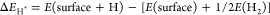
Then, the free energy of H adsorption, Δ*G*_H*_, was calculated according to the following equation:
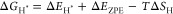
where Δ*E*_H*_, Δ*E*_ZPE_, and Δ*S*_H_ represent the changes of electronic energy, zero-point energy, and entropy from a gas phase to an adsorbed hydrogen, respectively. The climbing-image nudged-elastic-band (CI-NEB) method was used to locate the transition structures, and frequency calculations were used to verify the transition states.^28^

## Results and Discussion

Compared to the traditional MS_2_ containing a single type of metal, designing a pyrite structure with multiple bimetallic sites (e.g., M_a_M_b_S_2_) can significantly improve the catalytic performance, possibly because the modified electronic structure can significantly reduce the kinetic energy barrier in the HER process.^29,30^ Notably, most works reported their performances for HER in acidic electrolytes, where protons are abundant for hydrogen generation. However, the efficiency of water splitting is hindered by the sluggish kinetics in the oxygen evolution reaction (OER) in the acidic electrolytes, and hence, it is usually carried out in the alkaline electrolytes for improving the OER efficiency. In this regard, the catalytic property and durability of the transition metal pyrite electrocatalyst for HER in alkaline electrolytes are important and rarely discussed. We aimed to have deep insights into the course with stabilizer-free NiS_2_, CoS_2_, and Ni_0.5_Co_0.5_S_2_ hollow microspheres as the electrocatalysts for HER in the KOH electrolyte (pH = 13.8 ± 0.1) and carried out in operando spectroscopic investigation for understanding the mechanistic story.

**Figure 2:**
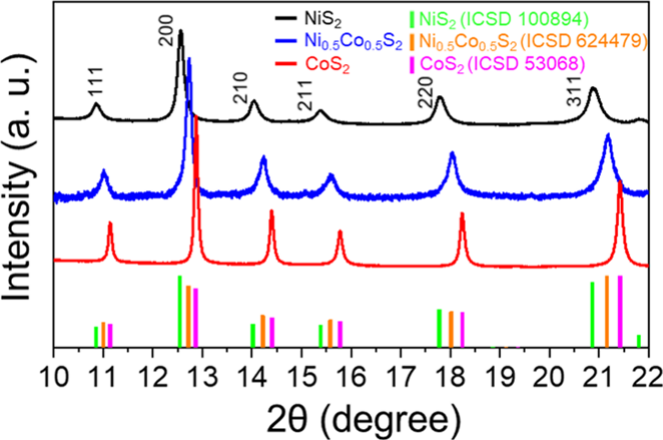
Synchrotron X-ray diffraction patterns of synthesized NiS_2_, Ni_0.5_Co_0.5_S_2_, CoS_2_, and their reference patterns from ICSD.

The samples of stabilizer-free NiS_2_, CoS_2_, and Ni_0.5_Co_0.5_S_2_ were prepared by the hydrothermal method as described in Experimental Section. Figure 2 shows the powder X-ray diffraction (PXRD) patterns of the resulting samples obtained with an X-ray energy of 20 keV (λ = 0.61992 Å). All three patterns have strong feature peaks of (111), (200), (210), (211), (220), and (311), which indicates the pyrite structures corresponding with NiS_2_ (ICSD 100894), Ni_0.5_Co_0.5_S_2_ (ICSD 624479), and CoS_2_ (ICSD 53068). Figure S1 and Table S1 show the results of Rietveld refinements for their PXRD patterns upon the reference ICSD patterns in which *R*_wp_ infers the weighted profile fitting agreement factor. All the *R*_wp_ values are lower than 5%, meaning very low difference between the experimental and reference patterns and, therefore, the high purities in the crystal structures. It is worth noting that the feature peaks of Ni_0.5_Co_0.5_S_2_, i.e., (111) at 11.02°, (200) at 12.74°, and (210) at 14.24°, are all in-between those of NiS_2_ and CoS_2_. According to the results of Rietveld refinements in Figure S1 and Table S1, the lattice constants of NiS_2_, Ni_0.5_Co_0.5_S_2_, and CoS_2_ unit cells are 5.675, 5.600, and 5.532 Å, respectively. Given the order in the lattice constants (NiS_2_ > Ni_0.5_Co_0.5_S_2_ > CoS_2_), the Ni–Co blended pyrite structure in the sample of Ni_0.5_Co_0.5_S_2_ is verified. To further determine the accurate atomic ratios among N, Co, and S in the samples, we performed inductively coupled plasma-mass (ICP-MS) spectroscopy measurements and obtained their average chemical formulas as Ni_1.02_S_2.00_, Ni_0.55_Co_0.48_S_2.00_, and Co_0.93_S_2.00_ by normalizing with the sulfur molar numbers (Table S2).

**Figure 3:**
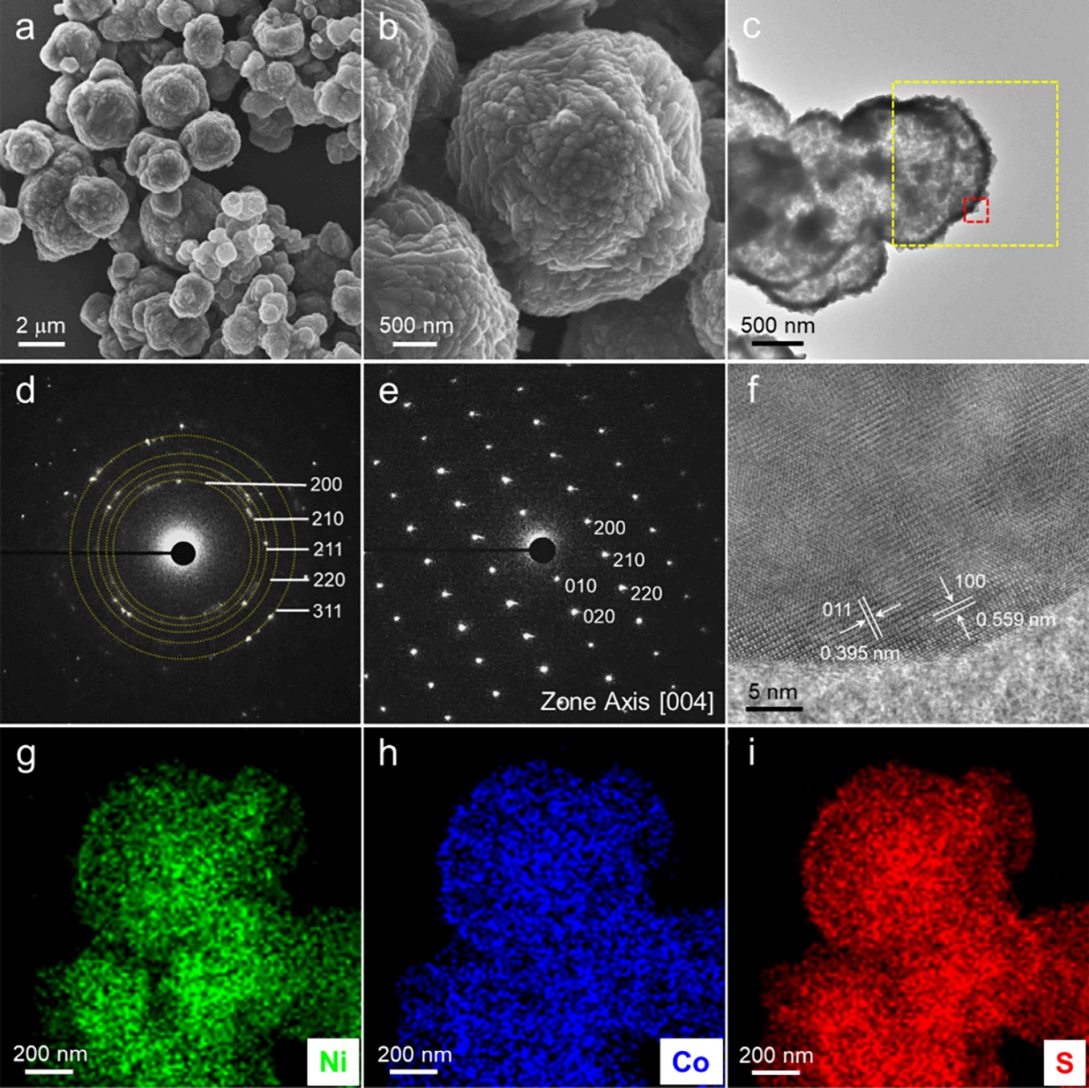
(a) Low- and (b) high-magnification SEM images of Ni_0.5_Co_0.5_S_2_ nanocrystal-assembled microspheres. (c) Bright-field TEM image of Ni_0.5_Co_0.5_S_2_ microspheres and (d, e) corresponding SAED patterns for (d) the yellow- and (e) red-marked areas. (f) High-resolution TEM image of the red-marked area in panel (c). (g–i) STEM-EDX maps of (g) Ni, (h) Co, and (i) S distributions.

Figure 3 shows the results of structural analyses of Ni_0.5_Co_0.5_S_2_ microspheres. From the SEM images in Figure 3a,b, the synthesized Ni_0.5_Co_0.5_S_2_ sample formed microspheres with a diameter of 2.0 ± 0.7 μm (Figure S5a) and very rough surfaces. The bright-field TEM image in Figure 3c shows that the microspheres are hollow in the interiors. The hollow structures can be observed clearly in the high-angle annular dark-field (HAADF) images as well as in Figure S4a. To realize the hollow microstructure, selected-area electron diffraction (SAED) was performed over the whole sphere (yellow-marked area) and a tip on the sphere (red-marked area) as shown in Figure 3c. As a result, Figure 3d obtained from the yellow-marked area shows the ring pattern, revealing that the microspheres are polycrystalline. The diffraction rings correspond with the cubic pyrite containing {200}, {210}, {211}, {220}, and {311} crystal faces. In contrast, the spot pattern in Figure 3e reflects that the rough tips on the microsphere are single-crystalline pyrite, which is also verified by the ordered lattice fringes in the tip in Figure 3f. The maps of STEM-EDX display homogeneous distributions of Ni, Co, and S elements over the microspheres (Figure 3g–i). According to the results, it is rational to assume that the formation of Ni_0.5_Co_0.5_S_2_ microspheres is attributed to reconstruction (assembling along with aggregation) of Ni_0.5_Co_0.5_S_2_ nanocrystals without a capping agent. Similar to the sample of Ni_0.5_Co_0.5_S_2_, those of NiS_2_ and CoS_2_ also formed microspheres with diameters of 2.5 ± 0.5 and 1.5 ± 0.5 μm, respectively (Figure S5b,c). The bright-field TEM images in Figures S2c and S3c display the hollow structures for NiS_2_ and CoS_2_ microspheres. The maps of STEM-EDX reveal that the microspheres are composed of NiS_2_ (Figure S2g–i) and CoS_2_ (Figure S3g–i), and their accurate compositions are Ni_1.02_S_2.00_ and Co_0.93_S_2.00_ determined by ICP-MS (Table S1). The SAED patterns (Figures S2d,e and S3e) and TEM images (Figures S2f and S3d,f) infer that the formation of NiS_2_ and CoS_2_ microspheres is also attributed to nanocrystal aggregation. However, a majority of NiS_2_ nanocrystals are nanocubes bounded by six {100} crystal faces (Figure S4b), while most CoS_2_ nanocrystals are nanooctahedra with eight {111} crystal faces on the surface (Figure S3b), indicating the apparently different crystal faces exposed on the NiS_2_ and CoS_2_ microsphere surfaces.^31^Figure S6 shows the XPS results of Ni 2p (Figure S6a,b), Co 2p (Figure S6c,d), and S 2p (Figure S6e–g) for examining the surface states of hollow NiS_2_, CoS_2_, and Ni_0.5_Co_0.5_S_2_ microspheres, respectively. In the spectra of Ni 2p, Ni^2+^ is the sole valence state over the NiS_2_ (2p_3/2_ = 854.14 eV) and Ni_0.5_Co_0.5_S_2_ (2p_3/2_ = 854.07 eV) surfaces. However, those of Co 2p reveal the coexistence of Co^2+^ and Co^3+^ over the CoS_2_ (2p_3/2_ 780.38 eV for Co^2+^ and 854.14 eV for Co^3+^) and Ni_0.5_Co_0.5_S_2_ (2p_3/2_ 779.97 eV for Co^2+^ and 854.07 eV for Co^3+^) surfaces. It hints that the role of Co is likely more active than Ni in the pyrite structures under ambient condition. Figure S6e–g presents S 2p for NiS_2_, CoS_2_, and Ni_0.5_Co_0.5_S_2_ in order. The results reveal the similarity in their valence states of sulfur where the low binding energy regions (ca. 162.93 eV) represent the surface S_2_^–^ resulting from the relaxation of S^–^ after breaking S–S bonds on the pyrite surface. The peaks around 163.5 eV indicate the presence of polysulfide.^32^

**Figure 4:**
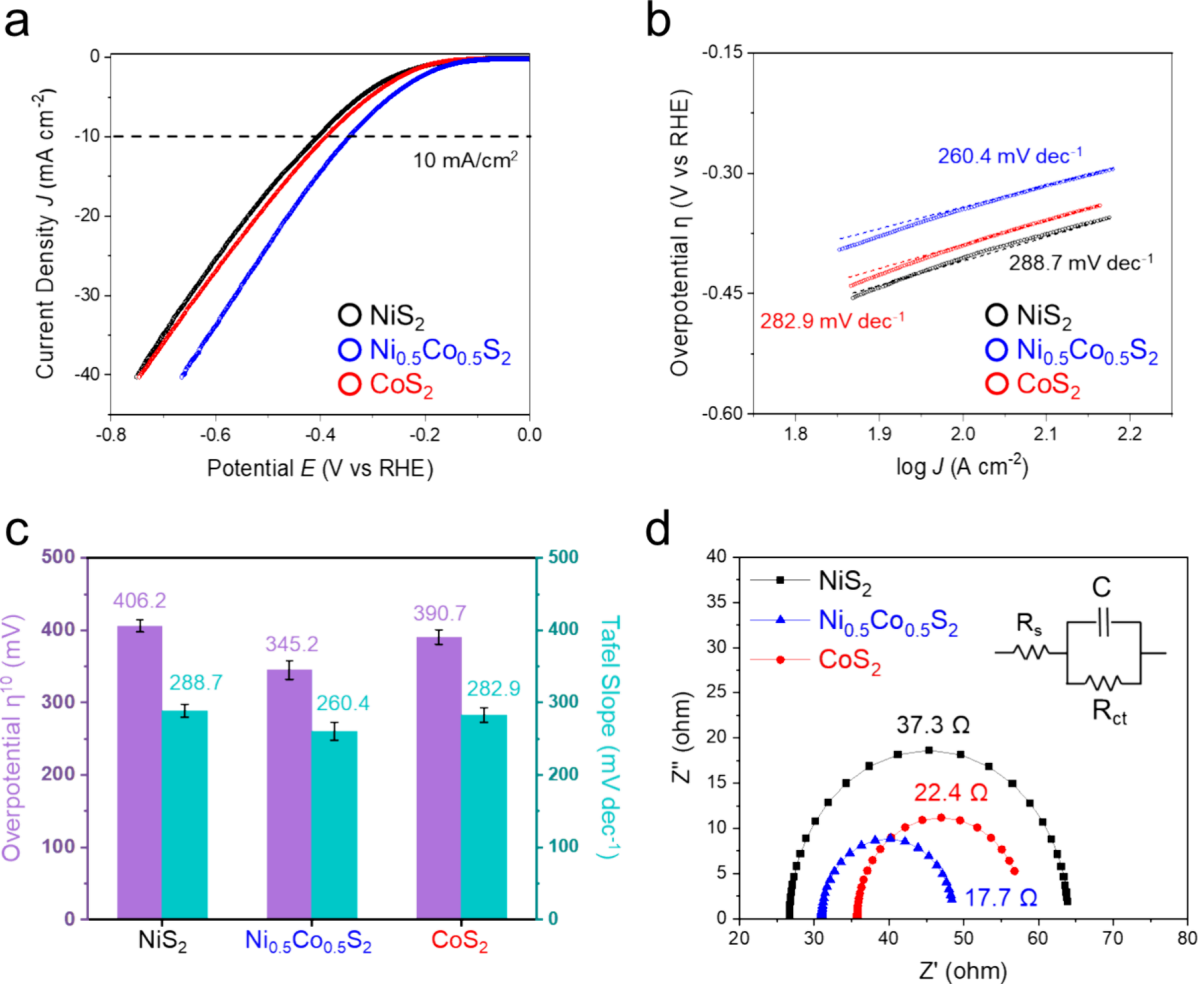
(a) HER LSV polarization curves, (b) Tafel plots, (c) summarized column charts of overpotentials and Tafel slopes, and (d) Nyquist plots for the hollow microspheres of NiS_2_, Ni_0.5_Co_0.5_S_2_, and CoS_2_.

Since the microspheres with well-defined crystal structures and compositions were obtained, the comparison experiments of microsphere-catalyzed HER in the alkaline electrolyte of KOH turned out feasible. The details of electrode preparations, electrochemical measurements, and in operando spectroscopic analyses were described in Experimental Section. Figure S7 shows the CVs scanned at the open-circuit potentials (OCPs) at different scan rates (*r*) of 10, 20, 30, 40, 50, 60, 80, and 100 mV/s for Ni_0.5_Co_0.5_S_2_ (Figure S7a), NiS_2_ (Figure S7c), and CoS_2_ (Figure S7e). From their corresponding plots of Δ*I*_OCP_/2 vs *r*, where Δ*I*_OCP_ = *I*_top_ – *I*_bottom_ at OCP, the obtained slopes denote the values of *C*_dl_ for the samples, which are 0.210 mF for Ni_0.5_Co_0.5_S_2_ (Figure S7b), 0.122 mF for NiS_2_ (Figure S7d), and 0.156 mF for CoS_2_ (Figure S7f). The values of their electrochemically active surface areas (ECSAs) hence are 5.25, 3.05, and 3.90 cm^–2^ for Ni_0.5_Co_0.5_S_2_, NiS_2_, and CoS_2_, respectively, determined by the equation *C*_dl_/*C*_s_ (see Experimental Section). Figure 4a shows the polarization curves of linear scan voltammetry (LSV) for water reduction catalyzed by NiS_2_ (black), Ni_0.5_Co_0.5_S_2_ (blue), and CoS_2_ (red) electrocatalysts, and Figure S8 shows the SEM images of the hollow microspheres after LSV. In the SEM images, there is no significant change in their morphologies. The LSV curves demonstrate that both the onset potentials and overpotentials (η^10^) at 10 mA/cm^2^ are in the order of Ni_0.5_Co_0.5_S_2_ < CoS_2_ < NiS_2_. The values of overpotentials (η^10^) are 345.2 mV for Ni_0.5_Co_0.5_S_2_, 390.7 mV for CoS_2_, and 406.2 mV for NiS_2_. The Tafel slopes mean how fast the current density increases against overpotential, which reflects the kinetics of microsphere-catalyzed HER. In Figure 4b, the values of Tafel slopes were calculated upon the overpotentials at 10 mA/cm^2^ in the LSV curves of Ni_0.5_Co_0.5_S_2_, NiS_2_, and CoS_2_. They are 260.4 mV dec^–1^ for Ni_0.5_Co_0.5_S_2_, 288.7 mV dec^–1^ for NiS_2_, and 282.9 mV dec^–1^ for CoS_2_, indicating that the kinetics of NiCoS_2_-catalyzed HER is the best among the three kinds of samples. All values of overpotentials and Tafel slopes are summarized in Figure 4c. The high values of Tafel slopes were caused by the initial water dissociation process in the Volmer step of the alkaline HER, which supplies H* to the following steps by cleaving the H–O–H bond, so that the reaction path of alkaline HER has larger energy barriers, and hence, it reduced the catalytic efficiency of HER.^33,34^ Nevertheless, the Ni_0.5_Co_0.5_S_2_-catalyzed HER rate is faster than the other two, and it manifests the possible benefits from the electronic effect between Ni and Co. Electrochemical impedance spectroscopy (EIS) provides the Nyquist plots of the Ni_0.5_Co_0.5_S_2_, NiS_2_, and CoS_2_ electrocatalysts at −0.4 V vs RHE for advanced exploration of the electrode kinetics in the HER (Figure 4d). The semicircle diagrams are obtained through an equivalent circuit (the inset in Figure 4d) where *R*_s_ denotes the internal resistance of the electrodes and electrolyte, *R*_ct_ means the charge-transfer resistance over the interface between the working electrode and the electrolyte, and *C* means the capacitance of the electrochemical system. The values of *R*_ct_ were calculated from the diameters of the semicircles. The smaller the diameter, the faster is the electron charge transfer. Ni_0.5_Co_0.5_S_2_ has the lowest *R*_ct_ value of 17.7 Ω compared to those of NiS_2_ (37.3 Ω) and CoS_2_ (22.4 Ω). This result represents better conductivity on the Ni_0.5_Co_0.5_S_2_ electrode and interprets its relatively higher HER efficiency among the three kinds of electrocatalysts.

**Figure 5:**
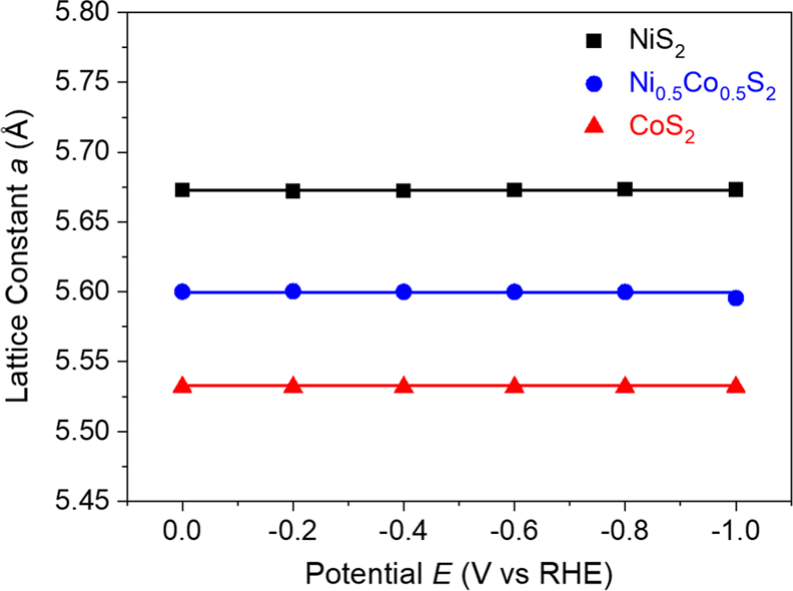
Relationship of lattice constant *a* vs potential *E* for the hollow NiS_2_, Ni_0.5_Co_0.5_S_2_, and CoS_2_ microspheres.

**Figure 6:**
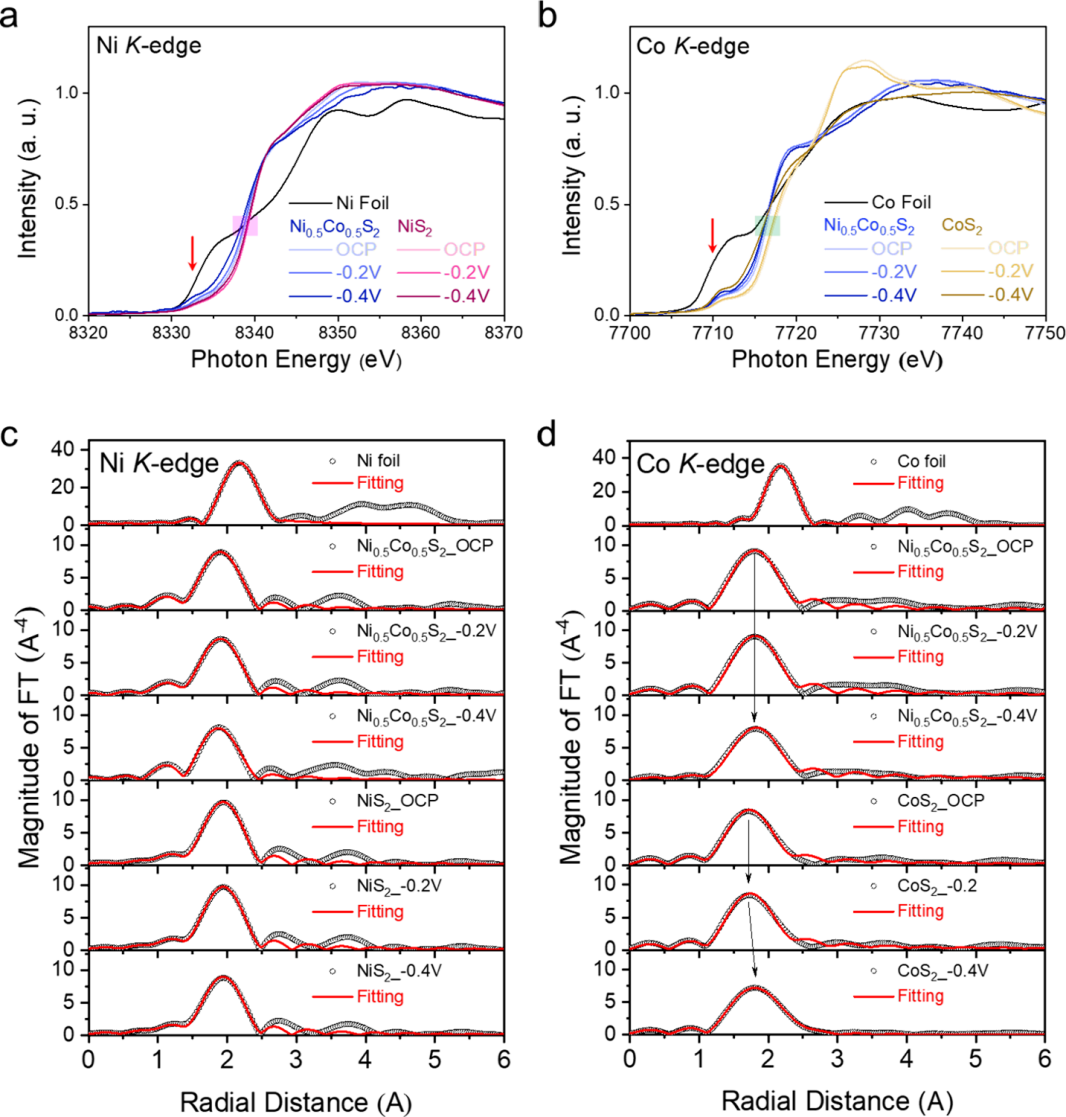
(a, b) XANES spectra and (c, d) *k*^3^-weighted Fourier transform EXAFS spectra of (a, c) Ni K-edges for Ni_0.5_Co_0.5_S_2_ and NiS_2_ and (b, d) Co K-edges for Ni_0.5_Co_0.5_S_2_ and CoS_2_.

Figure 5 is the plot showing the lattice constants (*a*) of hollow Ni_0.5_Co_0.5_S_2_, NiS_2_ microspheres, and CoS_2_ microspheres under different applied potentials (*E*). It was obtained from the summarized information on all their PXRD patterns after Rietveld refinements (Figures S9–S11 and Tables S3–S5). As a result, the values of *a* for all samples do not have a significant change with the applied potentials. For example, the *a* value of Ni_0.5_Co_0.5_S_2_ begins with 5.673 Å and ends up about the same at −0.1 V after experiencing different negative potentials. The cases of NiS_2_ and CoS_2_ maintain their *a* values, too. The results mean the good stability in the Ni_0.5_Co_0.5_S_2_, NiS_2_ and CoS_2_ crystal structures undergoing HER in alkaline KOH. On the other hand, it is known that the electrocatalytic activities are usually determined by the electronic and atomic structures of the electrocatalysts. Hence, in operando X-ray absorption spectroscopy (XAS) was also carried out for getting more insights. The XAS technique determines the local ordering around the absorbing atoms in the catalysts.^35^ The X-ray absorption near-edge structure (XANES) region of XAS provides the electronic structure and local geometric information, and the extended X-ray absorption fine structure (EXAFS) region is used to obtain the detailed local atomic structure around the absorbing atoms, for example, the nearest neighboring atomic type, the number of atoms in the specific coordination shell, the structural disorder, and the interatomic distance. Figure 6a,b presents the XANES spectra of the Ni K-edge for Ni_0.5_Co_0.5_S_2_ and NiS_2_ and the Co K-edge for Ni_0.5_Co_0.5_S_2_ and CoS_2_. Ni and Co metal foils are also presented for reference. The transition-metal K-edge is associated with a dipole-allowed transition from the 1s core level to the unoccupied 4p-derived orbitals, and therefore, the intensity of absorption peaks is positively proportional to the amount of unoccupied orbital states. In Figure 6a,b, there are weak pre-edges (red arrows, around 8333 eV for Ni and 7710 eV for Co), which are attributed to the dipole-forbidden transition from 1s to 3d orbitals. Pre-edge absorption is allowed due to the combination of stronger 3d–4p mixing and overlap of the metal 3d orbitals with the 2p orbitals of the ligand from the crystal field. It implies that the local structure of crystal field symmetries was different or had a distortion when the intensity was changed. In Figure 6a, the XANES Ni edge for Ni_0.5_Co_0.5_S_2_ in the pink area slightly shifts from 8338.8 to 8338.4 eV, while those for NiS_2_ remain around 8338.9 eV from the condition of applied open-circuit potential (OCP, referring to Figure S7) to −0.4 V (vs RHE). On the other hand, the morphologies of EXAFS profiles and the intensities of pre-edges for Ni_0.5_Co_0.5_S_2_ also display limited variations and those for NiS_2_ stay almost the same. The results manifest that Ni of NiS_2_ is actually stable in its valence under negative potentials. However, Ni of Ni_0.5_Co_0.5_S_2_, possibly promoted by Co, exhibits significant valence variations with increasing negative potentials. In Figure 6b, the Co edge for Ni_0.5_Co_0.5_S_2_ in the green area slightly shifts from 7716.3 to 7716.1 eV from the OCP to −0.4 V, and the EXAFS profiles do not vary much as well as the pre-edge. In contrast, Co for CoS_2_ exhibits significant shifts where the XANES edge moves from 7717.1 eV at the OCP and 7716.7 eV at −0.2 V to 7715.8 eV at −0.4 V. Meanwhile, the EXAFS profile of CoS_2_ and the intensity of its Co pre-edge have obvious variation from −0.2 to −0.4 V. The distinct behaviors between the two kinds of Co XANES of Ni_0.5_Co_0.5_S_2_ and CoS_2_ reflect that Co of CoS_2_ is sensitive to the applied negative potentials, which exhibits very significant valence reduction from −0.2 to −0.4 V. In addition, the results of three samples confirm the existence of the electronic synergy between Ni and Co in Ni_0.5_Co_0.5_S_2_ that enhances the HER kinetics.

To get further insights into the story behind these K-edge variations, the EXAFS spectra were analyzed by Fourier transform (FT) and fitting was carried out to acquire more information about the coordination shell around the energy-absorbed Ni or Co atom. Figure 6c shows the *k*^3^-weighted Fourier transform EXAFS spectra of the Ni K-edge for the Ni foil, Ni_0.5_Co_0.5_S_2_, and NiS_2_ with their fitting results shown as the red lines. The structural parameters obtained from the fitting analyses are abstracted in Table S6. The fitting models for the scattering paths of Ni–Ni and Ni–S were from ICSD entries 37502 and 68167. The magnitude of FT in the spectra reflects the short-range structural order and crystal size, which becomes weaker with decreasing crystal size (lower coordination number) and increasing disorder in the crystal structure. The radial distance denotes the interatomic distance from the energy-absorbed atom. According to the results in Figure 6a and Table S6, the Ni–Ni bond distance of the Ni foil is around 2.48 Å. Neither the Ni–S bond distance (2.36 → 2.34 Å) nor the magnitude (8.92 → 8.10) in Ni_0.5_Co_0.5_S_2_ changes largely from OCP to −0.4 V. It indicates the stability of the Ni-coordinated shell under the negative potentials without Ni metal generation. The drop in the average coordination number of Ni–S from 5.86 to 4.42 infers little sulfur leaching over the catalyst surfaces. The same phenomenon is also observed in the case of NiS_2_, which maintains the Ni–S bond distance at 2.39 Å and the magnitude in the range of 9.7–9.0 Å. There is also a small drop in N from 6.00 to 5.54. In Figure 6d and Table S7, the fitting models for the Co–Co and Co–S scattering paths were ICSD 44989 and 53068, respectively. The Co–Co bond distance from the Co foil is 2.19 Å. It is worth noting that the Co–S bond distance in Ni_0.5_Co_0.5_S_2_ is the same from OCP to −0.4 V (2.30 Å), while that in CoS_2_ underwent a large change from 2.26 Å at −0.2 V to 2.15 Å at −0.4 V. The fitting curve of CoS_2_ has significant shifts to longer radial distance from −0.2 to −0.4 V as well. On the other hand, N of Co–S in Ni_0.5_Co_0.5_S_2_ just slightly drops from 5.31 to 4.47 from OCP to −0.4 V, but that in CoS_2_ decreases from 5.21 to 1.62. This large change means that the Co coordination shell varies. It turns out the Co–Co scattering path from Co foil is required for achieving perfect fitting, which denotes the generation of the Co metal at −0.4 V. However, it also reflects that the sizes of the generated Co metal crystal domains should be very limited (<2 nm) over the microsphere surfaces, which is out of the resolution of synchrotron X-ray diffraction, and therefore, the Co metal was not observed in the PXRD pattern.

In light of the results from HER, XRD, and XAS, it is worth considering whether the improved HER kinetics over Ni_0.5_Co_0.5_S_2_ in the KOH electrolyte has something to do with the exposed crystal faces on microsphere surfaces and the electronic structure. Thus, density functional theory (DFT) calculations of alkaline hydrogen evolution reaction (HER) activity across various transition metal pyrite surfaces were conducted to sort the puzzle out from the viewpoint of energy. Theoretical work by Nørskov et al.^36−38^ demonstrated that as inferred from the kinetic model, the HER activity strongly correlates with the free energy of H adsorption (Δ*G*_H*_) onto the electrocatalyst surface. Accordingly, Δ*G*_H*_ has been widely employed as a descriptor of the HER activity. In an alkaline environment, the HER involves an additional process of water adsorption and dissociation on the catalyst surface, followed by hydrogen (H*) adsorption, recombination, and subsequent desorption of molecular hydrogen (H_2_). Therefore, the barrier for water dissociation and the free energy of hydrogen adsorption were calculated using DFT to evaluate the alkaline HER activity of various transition metal pyrite surfaces in this work.

**Figure 7:**
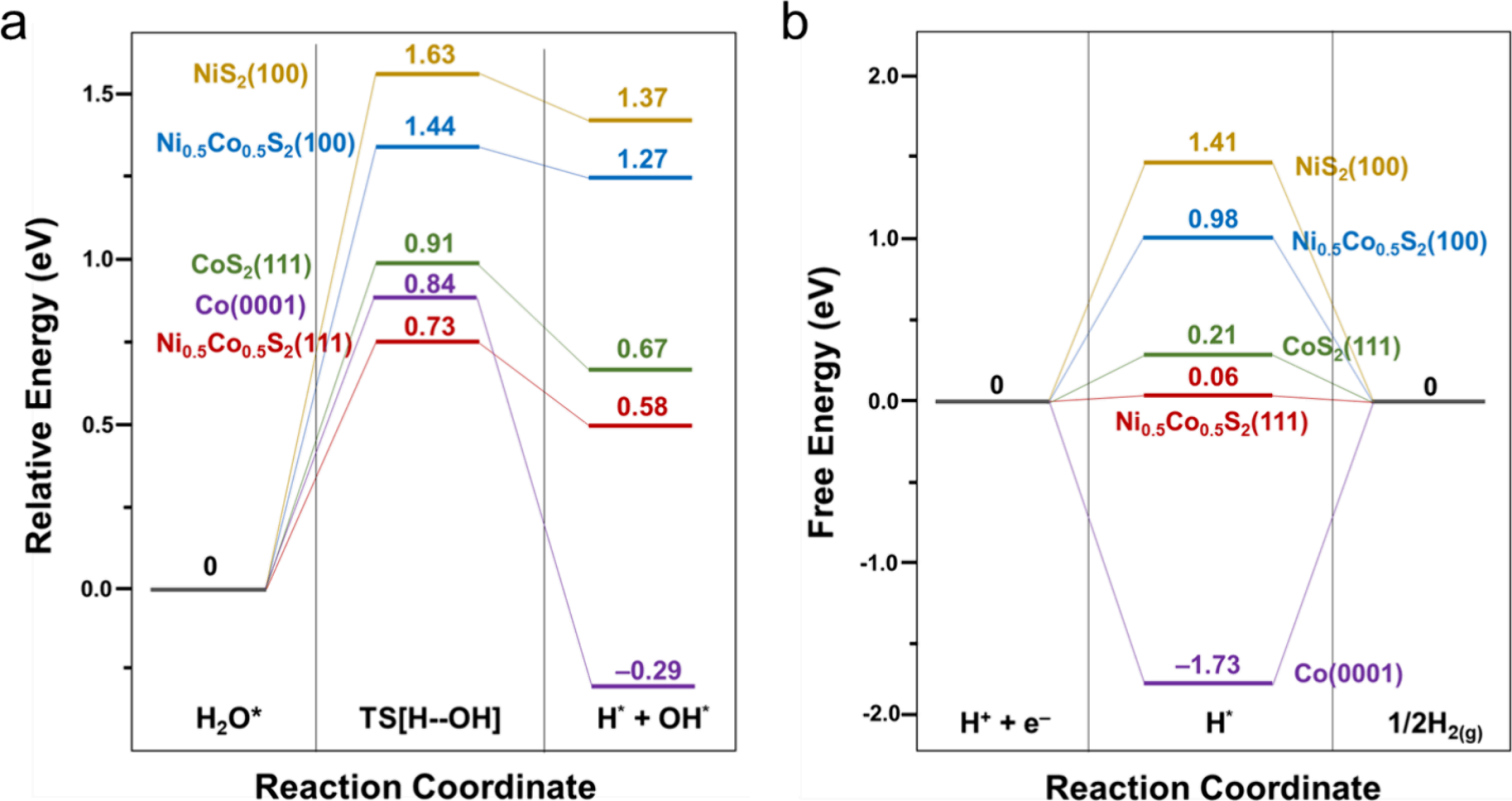
(a) Energy diagram for breaking the H–OH bond of H_2_O in the Volmer step on various surfaces. (b) Free energy diagram of HER (Δ*G*_H*_) on various surfaces.

Since we could not exactly tell which major crystal faces are exposed on the surfaces of Ni_0.5_Co_0.5_S_2_ microspheres like NiS_2_ and CoS_2_ by EM imaging, we constructed transition metal pyrite surfaces of Ni_0.5_Co_0.5_S_2_(100) and Ni_0.5_Co_0.5_S_2_(111) in addition to CoS_2_(111), NiS_2_(100), and Co(0001) to assess their HER activities, as illustrated in Figure S12. The energy diagram for breaking the H–OH bond of H_2_O in the Volmer step is shown in Figure 7a. In addition, the corresponding structures of the initial, transition, and final states on various surfaces are shown in Figure S13. The energy barriers for H–OH bond dissociation are 0.73, 0.91, 1.44, and 1.63 eV on the Ni_0.5_Co_0.5_S_2_(111), CoS_2_(111), Ni_0.5_Co_0.5_S_2_(100), and NiS_2_(100) surfaces, respectively. This suggests higher HER activity in the Volmer step on the Ni_0.5_Co_0.5_S_2_(111) surface. To identify potentially stable adsorption sites for hydrogen (H) on these surfaces, we systematically placed H at various feasible locations. The most stable adsorption sites of H on various surfaces are illustrated in Figure S14, and the resulting free energy of H adsorption (Δ*G*_H*_) is presented in Figure 7b. DFT calculations reveal that the hydrogen adsorption free energies on NiS_2_(100), Ni_0.5_Co_0.5_S_2_(100), CoS_2_(111), and Ni_0.5_Co_0.5_S_2_(111) are 1.14, 0.98, 0.21, and 0.06 eV, respectively. Among these transition metal pyrite surfaces, the free energy of H adsorption on Ni_0.5_Co_0.5_S_2_(111) is the closest to the optimal Δ*G*_H*_ value (Δ*G*_H*_ = 0), indicating its higher HER activity in the Heyrovsky/Tafel step. These theoretical findings demonstrate a consistent trend in HER activity on transition metal pyrite surfaces in both the Volmer and Heyrovsky/Tafel steps, with Ni_0.5_Co_0.5_S_2_(111) showing the highest activity, followed by CoS_2_(111), Ni_0.5_Co_0.5_S_2_(100), and NiS_2_(100). This implies that the synthesized Ni_0.5_Co_0.5_S_2_ microspheres should have a majority of the {111} crystal faces exposed over the surfaces.

Given that the Co metal gradually forms on the CoS_2_(111) surface in the HER process, the HER activity on the Co(0001) surface (Co is assumed to have a hexagonal crystal structure) has also been investigated by DFT calculations. The result shows that the free energy of H adsorption on Co(0001) is −1.73 eV, indicating a strong interaction between H and the Co(0001) surface. This interaction leads to surface poisoning, ultimately resulting in a reduction in the HER activity of the Co(0001) surface.

## Conclusions

To unravel the story of the HER mechanism in the alkaline electrolyte, we synthesized three types of pyrite materials including those serving as the model electrocatalysts. These pyrite materials were stabilizer-free in the synthetic steps and ultimately formed the morphology of hollow microspheres caused by the reconstruction of their nanocrystals. In the HER experiments, we examined the HER efficiencies and kinetics of the pyrite NiS_2_, CoS_2_, and Ni_0.5_Co_0.5_S_2_ materials (electrocatalysts) in the alkaline electrolyte (KOH) and provided insights into their HER mechanisms through the cooperative analyses by in operando X-ray spectroscopies and density function theory (DFT) calculations. Both the values of Tafel slopes and charge-transfer resistances (*R*_ct_) are in the order NiS_2_ (288.7 mV dec^–1^, 37.3 Ω) > CoS_2_ (282.9 mV dec^–1^, 22.4 Ω) > Ni_0.5_Co_0.5_S_2_ (260.4 mV dec^–1^, 17.7 Ω). The results reveal that H_2_ evolution in the alkaline electrolyte could be significantly facilitated by the factor of Ni–Co blending in the pyrite structure, which is the target we aim to unravel. Figure 1 is the schematic hypothesis to support our observations on the improved H_2_ evolution by the hollow Ni_0.5_Co_0.5_S_2_ microspheres. On the surfaces of hollow NiS_2_ (100 faces in majority) and Ni_0.5_Co_0.5_S_2_ microspheres (111 faces in majority), the structures are stable in KOH under the applied potentials confirmed by in operando XRD and XAS spectroscopies. However, the surfaces of hollow CoS_2_ microspheres (111 faces in majority) undergo reduction and generate Co metallic crystal domains. From DFT calculations, the energy barrier in the first step of H_2_O adsorption/dissociation in alkaline HER is 0.73 eV and the free energy of the following hydrogen desorption (to form H_2_) is only 0.06 eV over the Ni_0.5_Co_0.5_S_2_ surface. In contrast, the values are 1.63 and 1.41 eV for the NiS_2_ surface, which indicate that Ni is obviously a much more inert active site for alkaline HER than Ni_0.5_Co_0.5_S_2_, although the NiS_2_ surface is electrochemically stable. For the partially reduced CoS_2_ surface, the values are 0.91 and 0.21 eV for CoS_2_, slightly less energy-favored for alkaline HER compared to the Ni_0.5_Co_0.5_S_2_ surface. Notably, the generation of Co metal sites would further deactivate H_2_ evolution due to the large free energy (−1.73 eV) for the hydrogen desorption, although it is only 0.84 eV in the first step of H_2_O adsorption/dissociation. In light of the results, we can demonstrate that H_2_ evolution over the Ni_0.5_Co_0.5_S_2_ surface is truly improved by the synergistic electronic effect of Ni and Co. We believe that our findings have provided the clear story about the HER mechanisms catalyzed by the pyrite materials in KOH and a guidance toward catalyst optimization, leading to improved water splitting.

## Supporting Information Available

Tables, SEM, TEM images, size-distribution histograms, CVs, PXRD patterns, XAS spectroscopy, and DFT calculations (PDF)
