# Supplementary material for: In Operando X-ray Spectroscopic and DFT Studies Revealing Improved H2 Evolution by the Synergistic Ni–Co Electron Effect in the Alkaline Condition
Source: ACS Appl Mater Interfaces. 2024 May 20;16(21):27329–38. doi: 10.1021/acsami.4c02613 (PMC11145584; doi:10.1021/acsami.4c02613)
Supplement: Supplementary file 1 — am4c02613_si_001.pdf [file am4c02613_si_001.pdf]

Supporting Information

In-Operando X-Ray Spectroscopic and DFT Studies Revealing Improved H_2 Evolution by the Synergistic Ni-Co Electron Effect in the Alkaline Condition

Lian-Ming Lyu,[†, §] Han-Jung Li,[†, §] Ren-Shiang Tsai,[§] Ching-Feng Chen,[△] Yu-Chung Chang,[§] Yu-Chun Chuang,[‡] Cheng-Shiuan Li,[#] Jeng-Lung Chen,[‡] Te-Wei Chiu,[*, △] and Chun-Hong Kuo[*, §, ○, ‡]

[§]*Department of Applied Chemistry, National Yang Ming Chiao Tung University, Hsinchu 300093, Taiwan*

[○]*Center for Emergent Functional Matter Science, National Yang Ming Chiao Tung University, Hsinchu 300093, Taiwan*

[△]*Department of Materials and Mineral Resources Engineering, Institute of Materials Science and Engineering, National Taipei University of Technology, Taipei 106344, Taiwan*

[‡]*National Synchrotron Radiation Research Center, Hsinchu 300092, Taiwan*

[#]*Green Energy and Environment Research Laboratories, Industrial Technology Research Institute, Hsinchu 310401, Taiwan*

Contact information of corresponding authors

*(T.-W. Chiu) E-mail: tewei@ntut.edu.tw

*(C.-H. Kuo) E-mail: chunhong@nycu.edu.tw

Number of Pages: 21, Number of Tables: 7, Number of Figures: 12

Figure S1. Rietveld refinement for the PXRD patterns of hollow (a) $Ni_{0.5}Co_{0.5}S_2$, (b) NiS_2, and (c) CoS_2 microspheres. ……………………………………………………………………...........4

Table S1. Crystallographic data of hollow NiS_2, $Ni_{0.5}Co_{0.5}S_2$, and CoS_2 microspheres. …………….5

Table S2. Measured atomic compositions of hollow $Ni_{0.5}Co_{0.5}S_2$, NiS_2, and CoS_2 microspheres by ICP-MS. ……………………………………………………………………………………..6

Figure S2. (a) Low- and (b) high-magnification SEM images of hollow NiS_2 microspheres. (c) The bright-field TEM image of hollow NiS_2 microspheres and (d, e) the corresponding SAED patterns for (d) the yellow- and (e) red-marked areas. (f) High-resolution TEM imaging over the re-marked area in (c). (g) The SEM image of hollow NiS_2 microspheres for EDX mapping, and the corresponding (h) Ni and (i) S elemental distributions. ……………………………………………………………………7

Figure S3. (a) Low- and (b) high-magnification SEM images of hollow CoS_2 microspheres. (c, d) The bright-field TEM image of hollow NiS_2 microspheres. (e) The corresponding SAED pattern and (f) high-resolution TEM image over the re-marked area in (d). (g) The SEM image of hollow CoS_2 microspheres for EDX mapping, and the corresponding (h) Co and (i) S elemental distributions. ………………………………………………………………………………………8

Figure S4. (a) HAADF image of hollow $Ni_{0.5}Co_{0.5}S_2$ microspheres. (b) NiS_2 nanocubes observed in the sample of hollow NiS_2 microspheres. …………………………………………………….. ..9

Figure S5. Size-distribution histograms of hollow (a) $Ni_{0.5}Co_{0.5}S_2$, (b) NiS_2, and (c) CoS_2 microspheres. ……………………………………………………………………………............10

Figure S6. XPS of (a) Ni 2p, (b) Co 2p, and (c) S 2p for hollow NiS_2, CoS_2, and $Ni_{0.5}Co_{0.5}S_2$ microspheres.…………………………………………………………………………………..11

Figure S7. CVs obtained by scanning at the OCP at different scan rates (*r*) of 10, 20, 30, 40, 50, 60, 80, and 100 mV/s and their corresponding plots of ΔI_{OCP} vs *r* for hollow (a, b) $Ni_{0.5}Co_{0.5}S_2$, (c, d) NiS_2, and (e, f) CoS_2 microspheres. …………………………………………………………12

Figure S8. SEM images of hollow (a) NiS_2, (b) CoS_2, and (c) $Ni_{0.5}Co_{0.5}S_2$ microspheres after linear scan voltammetry………………………………………………………………………………13

Figure S9. The plots of (a) micro μ strain, (b) R_{wp}, (c) Uiso Ni, (d) Uiso S, and (e) $R(F^2)$ for hollow NiS_2 microspheres at different applied potentials. ………………………………………………14

Figure S10. The plots of (a) micro m strain, (b) R_{wp}, (c) Uiso Ni, (d) Uiso Co, (e) Uiso S, and (f) $R(F^2)$ for hollow $Ni_{0.5}Co_{0.5}S_2$ microspheres at different applied potentials. ……………………………………………………………………………………...15

Figure S11. The plots of (a) micro m strain, (b) R_{wp}, (c) Uiso Co, (d) Uiso S, and (e) $R(F^2)$ for hollow CoS_2 microspheres at different applied potentials. …………………………………………………16

Table S3. Crystallographic data of hollow NiS_2 microspheres at different potentials in HER. …….17

Table S4. Crystallographic data of hollow $Ni_{0.5}Co_{0.5}S_2$ microspheres at different potentials in HER. …………………………………………………………………………………….. 18

Table S5. Crystallographic data of hollow CoS_2 microspheres at different potentials in HER. ……19

Table S6. Structural parameters obtained from Ni *K*-edge EXAFS fitting analysis for samples. … 20

Table S7. Structural parameters obtained from Co K-edge EXAFS fitting analysis for samples. …..21

Figure S12. Top and side views of various transition metal pyrite surfaces. The grey, blue and yellow spheres represent Ni, Co, and S atoms. ……………………………………………………………...22
Figure S13. Optimized structures of initial, transition, and final states for breaking the H-OH bond in the Volmer step on various surface. ………………………………………………………………23
Figure S14. Optimized structures of H adsorption on various surfaces. ……………………………24

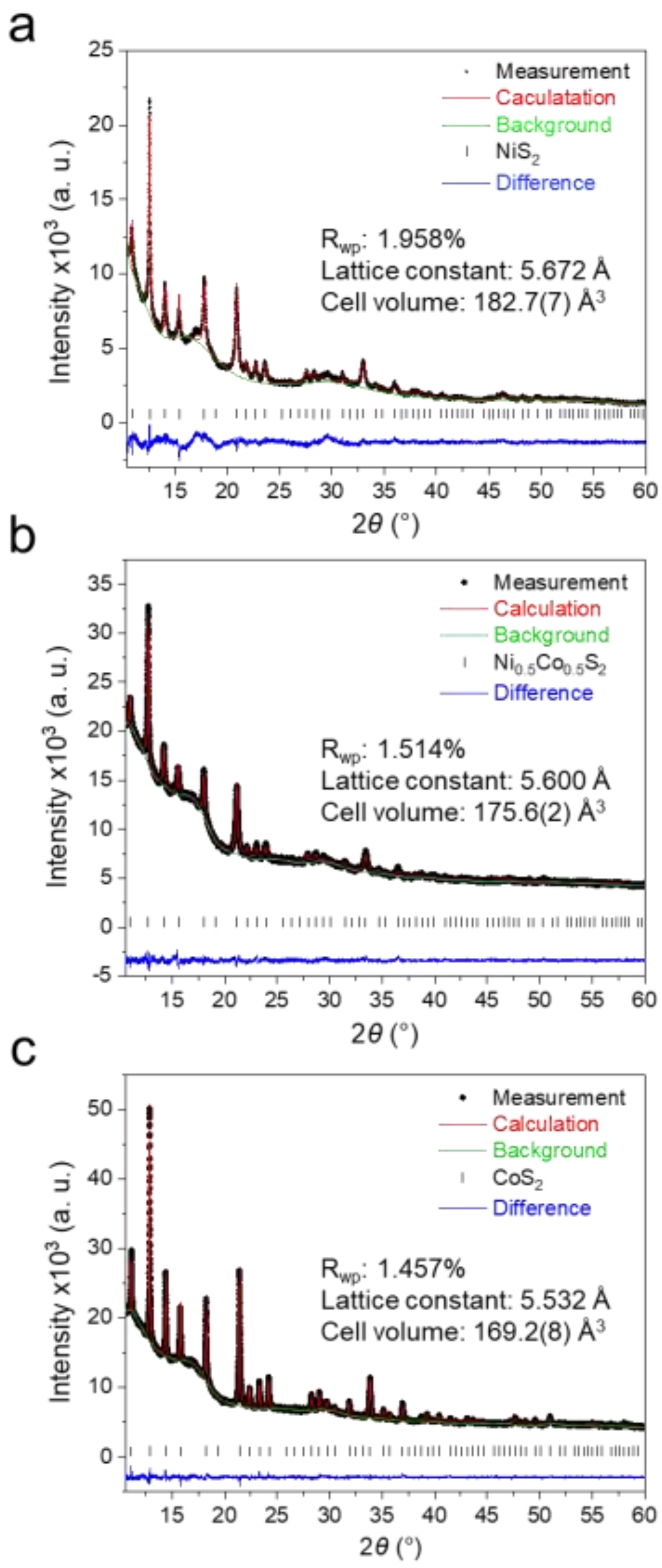

Figure S1. Rietveld refinement for the PXRD patterns of hollow (a) NiS_2, (b) $Ni_{0.5}Co_{0.5}S_2$, and (c) CoS_2 microspheres.

Table S1. Crystallographic data of hollow NiS_2, $Ni_{0.5}Co_{0.5}S_2$, and CoS_2 microspheres.

Formula	NiS_2	$Ni_{0.5}Co_{0.5}S_2$	CoS_2
FW(g/mol)	122.82	122.94	123.06
Space Group	P a -3		
λ (Å)	0.61992		
2θ (˚)	10.4 to 62.9	10.6 to 62.9	10.6 to 62.9
a (Å)	5.675(0)	5.600(1)	5.531(9)
V (Å^3)	182.7(7)	175.6(2)	169.2(9)
Micro μ Strain	27196	22526	10953
R_{wp}	1.958%	1.514%	1.457%
Zero Point Shift (˚)	0.0145	0.0154	0.0079
Uiso Ni (Å^2)	0.005(1)	0.007(1)	N/A
Uiso Co (Å^2)	N/A	0.007(2)	0.004(8)
Uiso S (Å^2)	0.003(4)	0.01	0.001(3)
R(F^2)	4.358%	8.001%	4.748%

Table S2. Measured atomic compositions of hollow $Ni_{0.5}Co_{0.5}S_2$, NiS_2, and CoS_2 microspheres by ICP-MS.

	Ni/ppm	Co/ppm	S/ppm	Formula
$Ni_{0.5}Co_{0.5}S_2$	25.60 ± 0.70	22.29 ± 0.63	50.57 ± 1.12	$Ni_{0.55}Co_{0.48}S_{2.00}$
NiS_2	75.26 ± 1.63	N/A	81.55 ± 3.48	$Ni_{1.02}S_{2.00}$
CoS_2	N/A	49.15 ± 0.59	57.53 ± 2.47	$Co_{0.93}S_{2.00}$

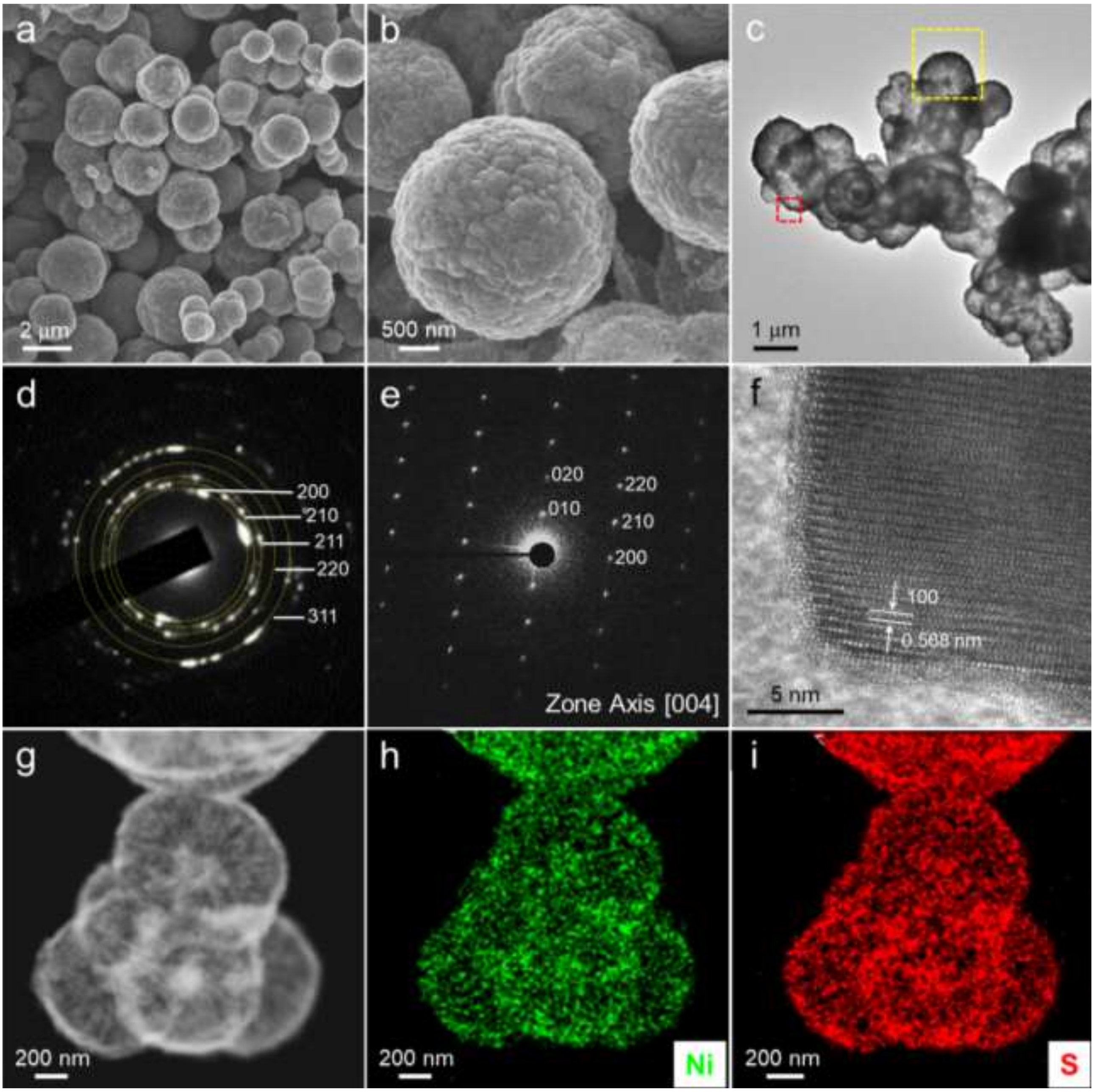

Figure S2. (a) Low- and (b) high-magnification SEM images of hollow NiS_2 microspheres. (c) The bright-field TEM image of hollow NiS_2 microspheres and (d, e) the corresponding SAED patterns for (d) the yellow- and (e) red-marked areas. (f) High-resolution TEM imaging over the re-marked area in (c). (g) The SEM image of hollow NiS_2 microspheres for EDX mapping, and the corresponding (h) Ni and (i) S elemental distributions.

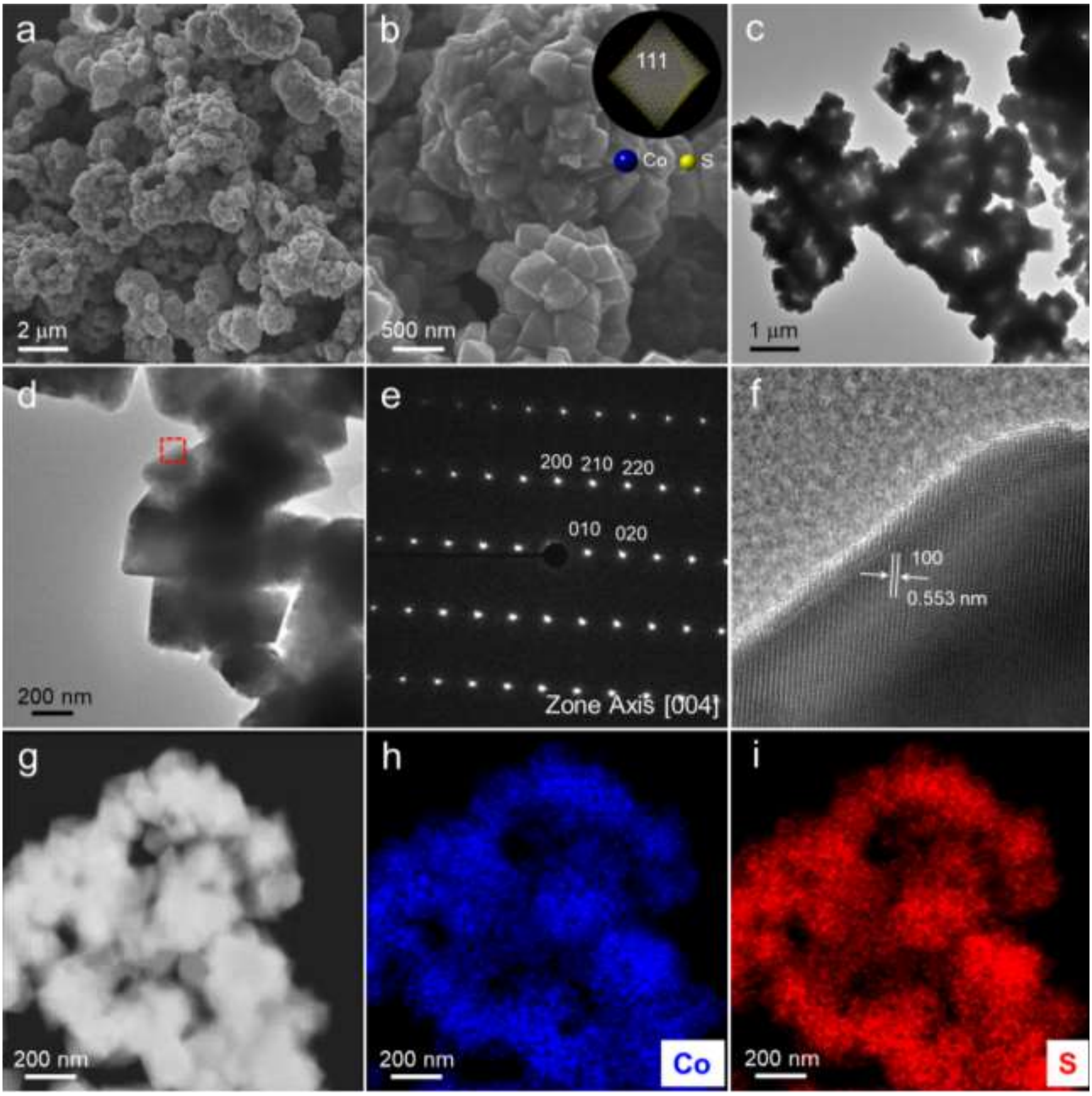

Figure S3. (a) Low- and (b) high-magnification SEM images of hollow CoS_2 microspheres. (c, d) The bright-field TEM image of hollow NiS_2 microspheres. (e) The corresponding SAED pattern and (f) high-resolution TEM image over the re-marked area in (d). (g) The SEM image of hollow CoS_2 microspheres for EDX mapping, and the corresponding (h) Co and (i) S elemental distributions.

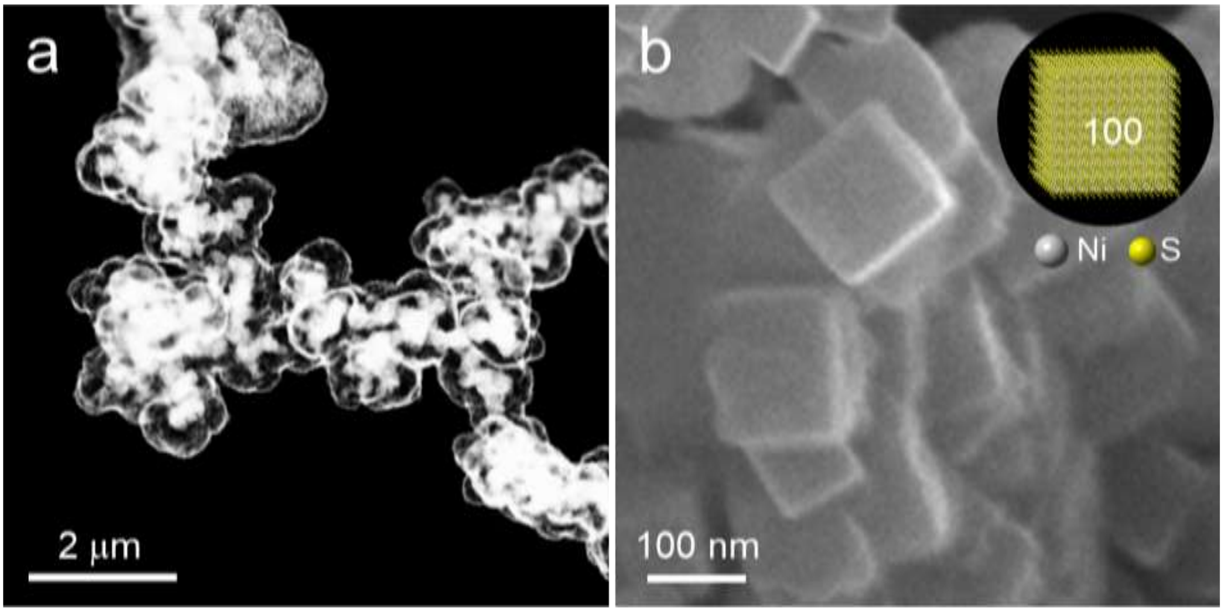

Figure S4. (a) HAADF image of hollow $Ni_{0.5}Co_{0.5}S_2$ microspheres. (b) NiS_2 nanocubes observed in the sample of hollow NiS_2 microspheres.

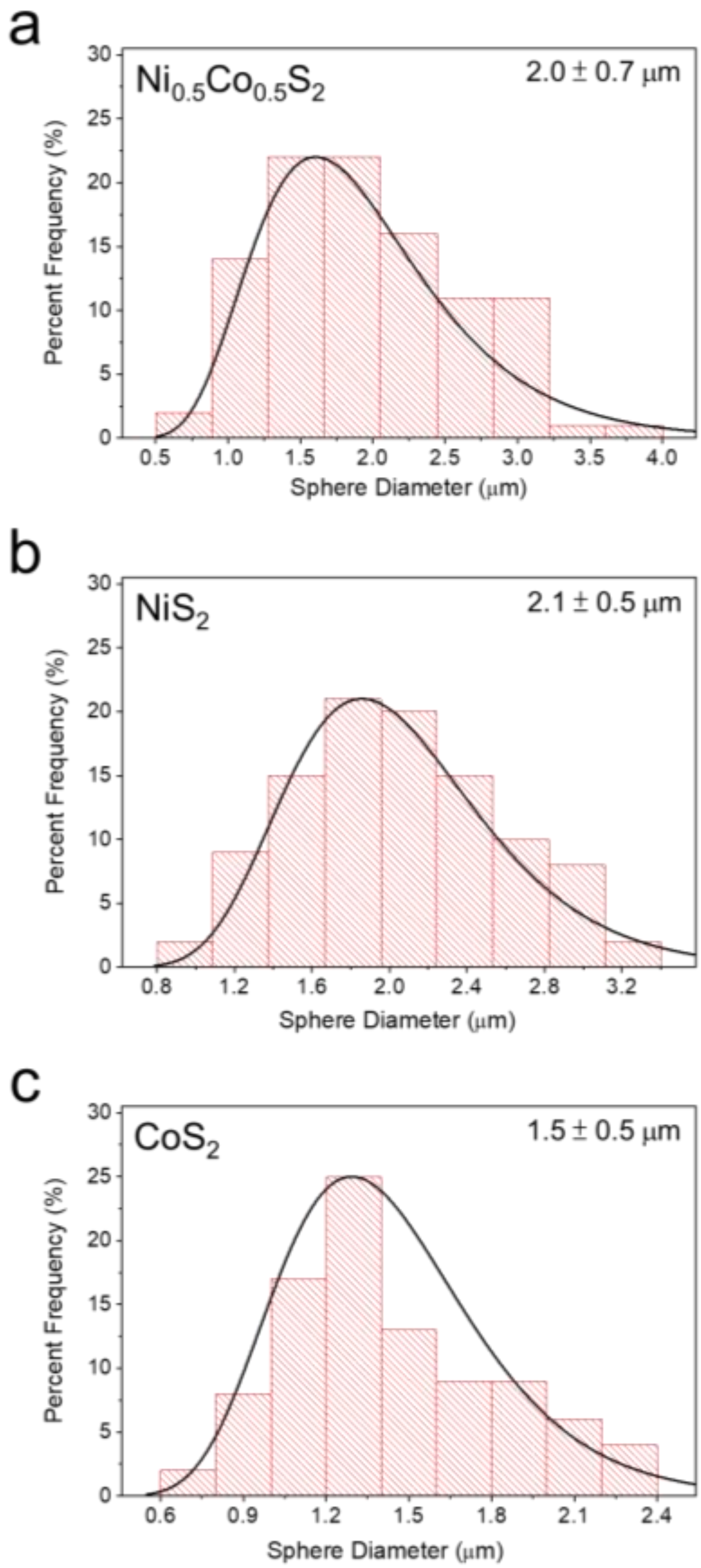

Figure S5. Size-distribution histograms of hollow (a) $Ni_{0.5}Co_{0.5}S_2$, (b) NiS_2, and (c) CoS_2 microspheres.

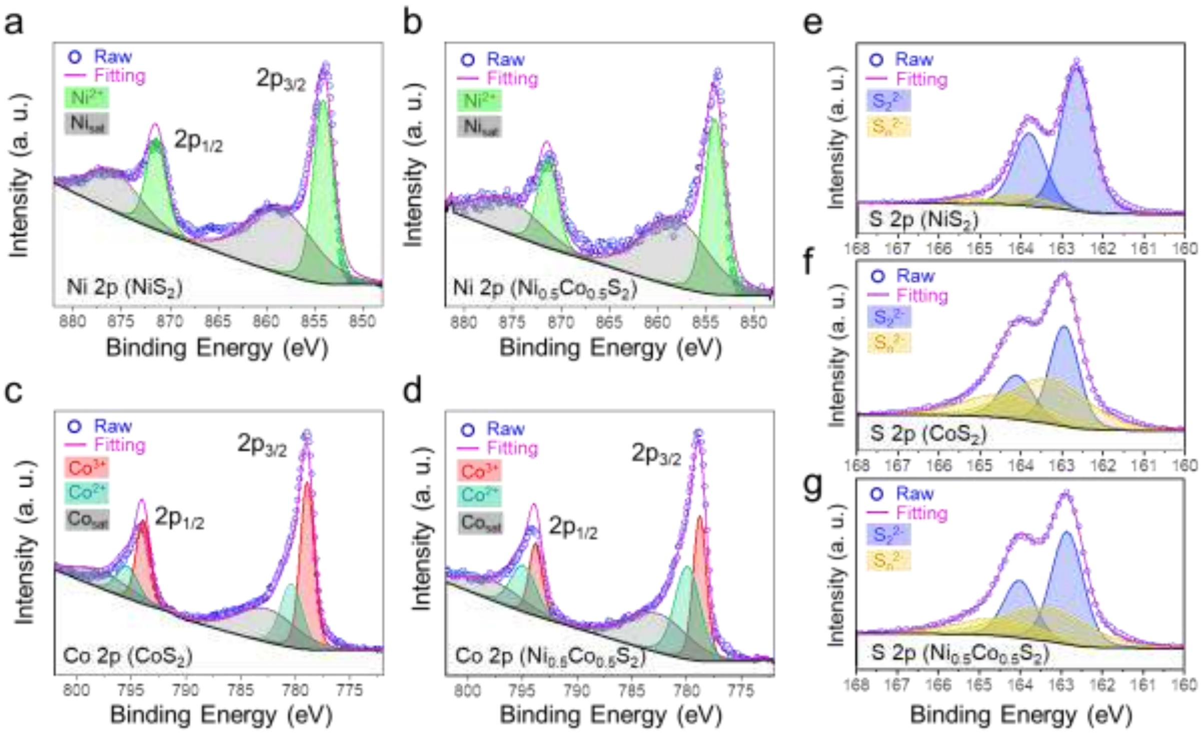

Figure S6. XPS of (a,b) Ni 2p, (c,d) Co 2p, and (e-g) S 2p for hollow NiS_2, CoS_2, and $Ni_{0.5}Co_{0.5}S_2$ microspheres.

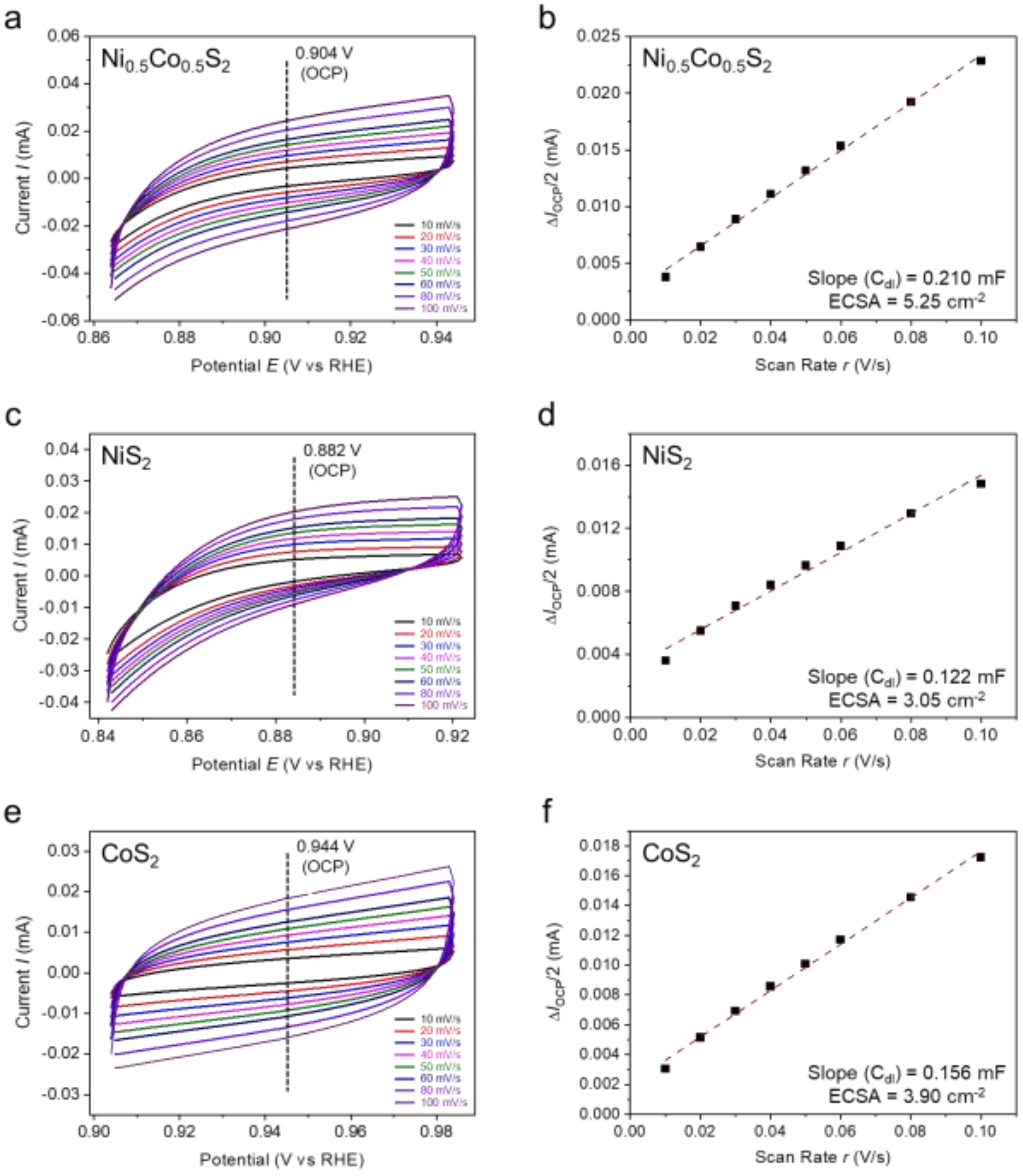

Figure S7. CVs obtained by scanning at the OCP at different scan rates (*r*) of 10, 20, 30, 40, 50, 60, 80, and 100 mV/s and their corresponding plots of ΔI_{OCP} vs *r* for hollow (a, b) $Ni_{0.5}Co_{0.5}S_2$, (c, d) NiS_2, and (e, f) CoS_2 microspheres.

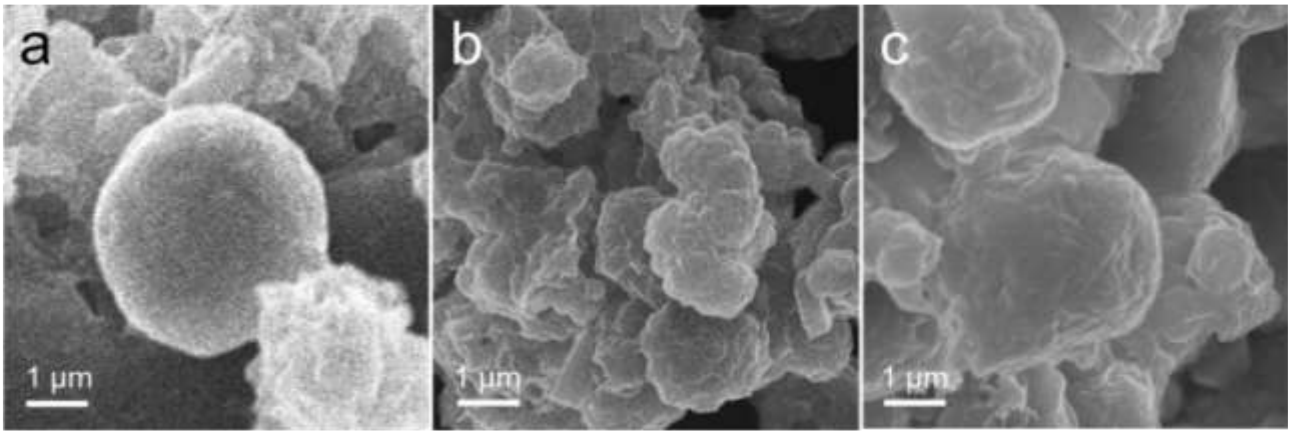

Figure S8. SEM images of hollow (a) NiS_2, (b) CoS_2, and (c) $Ni_{0.5}Co_{0.5}S_2$ microspheres after linear scan voltammetry.

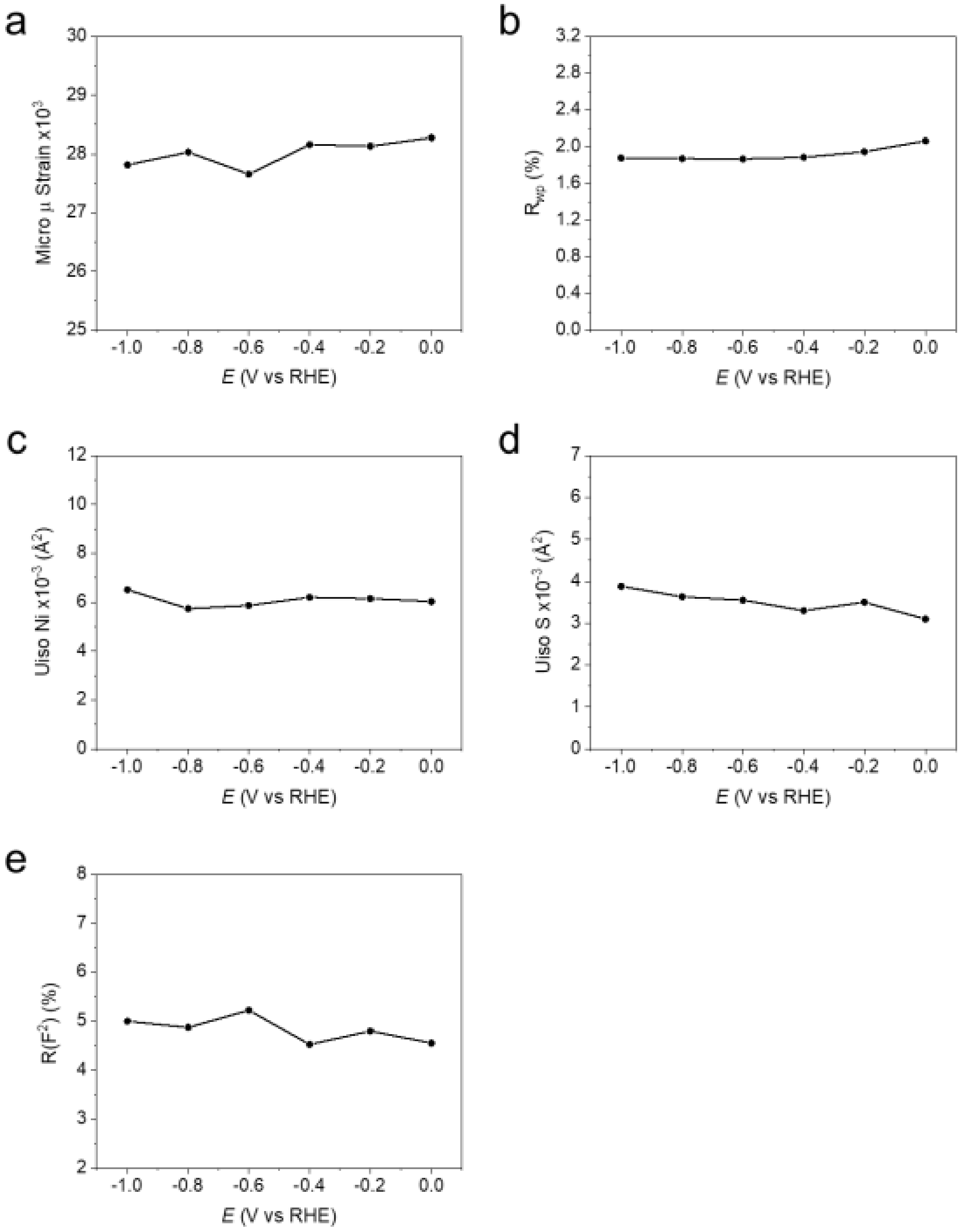

Figure S9. The plots of (a) micro μ strain, (b) R_{wp}, (c) Uiso Ni, (d) Uiso S, and (e) $R(F^2)$ for hollow NiS_2 microspheres at different applied potentials.

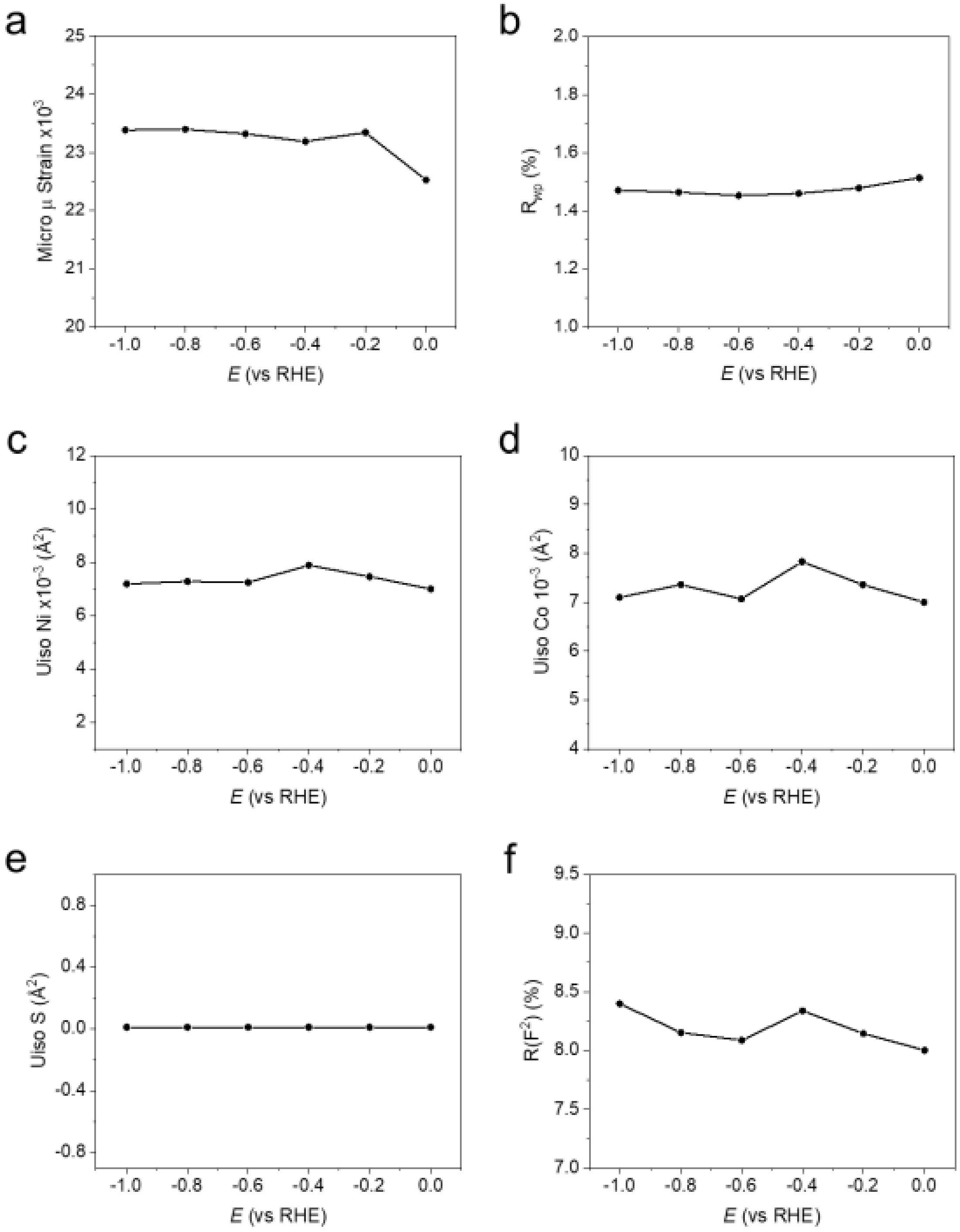

Figure S10. The plots of (a) micro μ strain, (b) R_{wp}, (c) Uiso Ni, (d) Uiso Co, (e) Uiso S, and (f) $R(F^2)$ for hollow $Ni_{0.5}Co_{0.5}S_2$ microspheres at different applied potentials.

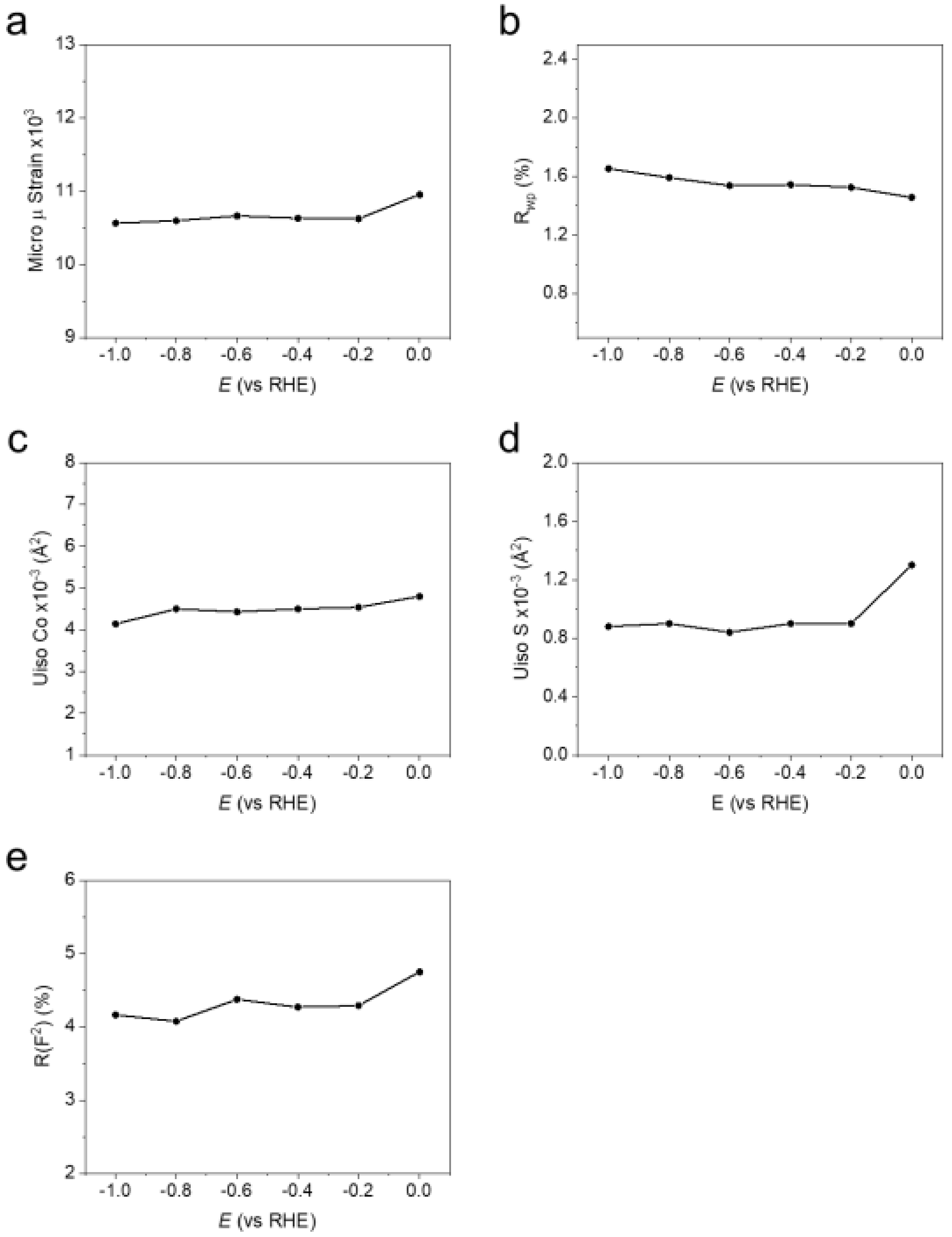

Figure S11. The plots of (a) micro μ strain, (b) R_{wp}, (c) Uiso Co, (d) Uiso S, and (e) $R(F^2)$ for hollow CoS_2 microspheres at different applied potentials.

Table S3. Crystallographic data of hollow NiS_2 microspheres at different potentials in HER.

Formula	NiS_2					
FW(g/mol)	122.82					
Space Group	P a -3 (205)					
λ (Å)	0.61992					
2θ (˚)	10.4 to 62.9					
E (V vs RHE)	no *E*	−0.2	−0.4	−0.6	−0.8	−1.0
a (Å)	5.674(8)	5.674(3)	5.675(3)	5.675(6)	5.675(9)	5.675(9)
V (Å^3)	182.7(5)	182.7(0)	182.8(0)	182.8(2)	182.8(5)	182.8(6)
Micro μ Strain	28275.9	28309.3	28161.4	27658.0	28033.7	27816.4
R_{wp}	2.064	1.934	1.885	1.867	1.871	1.878
Zero Point Shift (˚)	0.0144	0.0115	0.0139	0.0142	0.0144	0.0137
Uiso Ni (Å^2)	0.006(0)	0.005(0)	0.006(2)	0.005(8)	0.005(7)	0.006(5)
Uiso S (Å^2)	0.003(1)	0.003(5)	0.003(3)	0.003(5)	0.003(6)	0.003(8)
R(F^2)	4.549%	4.495%	4.522%	5.220%	4.871%	4.997%

Table S4. Crystallographic data of hollow $Ni_{0.5}Co_{0.5}S_2$ microspheres at different potentials in HER.

Formula	$Ni_{0.5}Co_{0.5}S_2$					
FW(g/mol)	122.94					
Space Group	P a -3 (205)					
λ (Å)	0.61992					
2θ (˚)	10.6 to 62.9					
E (V vs RHE)	no *E*	−0.2	−0.4	−0.6	−0.8	−1.0
a (Å)	5.600(0)	5.600(4)	5.600(0)	5.600(0)	5.599(9)	5.595(4)
V (Å^3)	175.6(2)	175.6(5)	175.6(1)	175.6(1)	175.6(1)	175.1(9)
Micro μ Strain	23059.0	23336.4	23189.0	23314.3	23391.2	23378.3
R_{wp}	1.534	1.479	1.460	1.453	1.464	1.471
Zero Point Shift (˚)	0.0154	0.0169	0.0148	0.0151	0.0144	0.0025
Uiso Ni (Å^2)	0.007(1)	0.007(4)	0.007(8)	0.007(2)	0.007(3)	0.007(1)
Uiso Co (Å^2)	0.007(1)	0.007(3)	0.007(8)	0.007(0)	0.007(2)	0.007(1)
Uiso S (Å^2)	0.010(0)	0.010(0)	0.010(0)	0.010(0)	0.010(0)	0.010(0)
R(F^2)	8.040%	8.144%	8.337%	8.086%	8.151%	8.398%

Table S5. Crystallographic data of hollow CoS_2 microspheres at different potentials in HER.

Formula	CoS_2					
FW(g/mol)	123.06					
Space Group	P a -3 (205)					
λ (Å)	0.61992					
2θ (˚)	10.6 to 62.9					
E (V vs RHE)	no *E*	−0.2	−0.4	−0.6	−0.8	−1.0
a (Å)	5.531(9)	5.531(8)	5.531(8)	5.531(8)	5.531(8)	5.531(9)
V (Å^3)	169.2(8)	169.2(7)	169.2(8)	169.2(7)	169.2(8)	169.2(8)
Micro μ Strain	10953.8	10624.3	10629.8	10662.5	10596.5	10563.9
R_{wp}	1.457	1.526	1.544	1.538	1.593	1.754
Zero Point Shift (˚)	0.0079	0.0079	0.0075	0.0076	0.008	0.0082
Uiso Co (Å^2)	0.004(8)	0.004(5)	0.004(4)	0.004(4)	0.004(4)	0.004(1)
Uiso S (Å^2)	0.001(3)	0.000(9)	0.000(9)	0.000(8)	0.000(9)	0.000(8)
R(F^2)	4.748%	4.287%	4.267%	4.372%	4.074%	4.160%

Table S6. Structural parameters obtained from Ni *K*-edge EXAFS fitting analysis for samples.

	Scattering Path	N	σ^2(Å^2)	R(Å)	R_f
Ni Foil	Ni-Ni	12	0.006	2.48	0.0001
$Ni_{0.5}Co_{0.5}S_2$ _ OCP	Ni-S	5.86	0.008	2.36	0.002
$Ni_{0.5}Co_{0.5}S_2$ _ –0.2 V	Ni-S	5.63	0.008	2.36	0.002
$Ni_{0.5}Co_{0.5}S_2$ _ –0.4 V	Ni-S	4.42	0.007	2.34	0.006
NiS_2 _ OCP	Ni-S	6	0.007	2.39	0.002
NiS_2 _ –0.2 V	Ni-S	6	0.007	2.39	0.001
NiS_2 _ –0.4 V	Ni-S	5.54	0.007	2.39	0.003

Fitting was done across the k range of 3.3 to 9.9 Å^{-1} and the R range of 1.0 to 2.4 Å for all samples. Where N is coordination number, R is distance between absorber and backscatter atoms, σ^2 is Debye–Waller factor value, R_f is R-factor characterizing the goodness of fitting. S_o^2 was fixed to 0.77 as determined from Ni foil. Error bounds (accuracies) characterizing the structural parameters obtained by EXAFS data analysis are estimated to be as follows: N, ±20%; R, ±1%; and σ^2, ±20%. Ni foil fitting model is from ICSD Code # 37502, and Ni-S fitting model is from NiS_2 ICSD Code # 68167.

Table S7. Structural parameters obtained from Co K-edge EXAFS fitting analysis for samples.

	Scattering Path	N	σ^2(Å^2)	R(Å)	R_f
Co Foil	Co-Co	12	0.006	2.49	0.0001
$Ni_{0.5}Co_{0.5}S_2$ _ OCP	Co-S	5.31	0.005	2.3	0.009
$Ni_{0.5}Co_{0.5}S_2$ _ –0.2 V	Co-S	5.12	0.005	2.3	0.01
$Ni_{0.5}Co_{0.5}S_2$ _ –0.4 V	Co-S	4.47	0.006	2.29	0.013
CoS_2 _ OCP	Co-S	5.21	0.006	2.26	0.009
CoS_2 _ –0.2 V	Co-S	4.91	0.005	2.26	0.01
CoS_2 _ –0.4 V	Co-S	1.62	0.003	2.15	0.004
	Co-Co	4.98	0.01	2.49	0.004

Fitting was done across the *k* range of 2.7 to 8.4 Å^{-1} and the *R* range of 1.0 to 2.5 Å for all samples. Where N is coordination number, R is distance between absorber and backscatter atoms, σ^2 is Debye–Waller factor value, R_f is R-factor characterizing the goodness of fitting. S_o^2 was fixed to 0.76 as determined from Co foil. Error bounds (accuracies) characterizing the structural parameters obtained by EXAFS data analysis are estimated to be as follows: N, ±20%; R, ±1%; and σ^2, ±20%. Co foil fitting model is from ICSD Code # 44989, and Co-S fitting model is from CoS_2 ICSD Code # 53068.

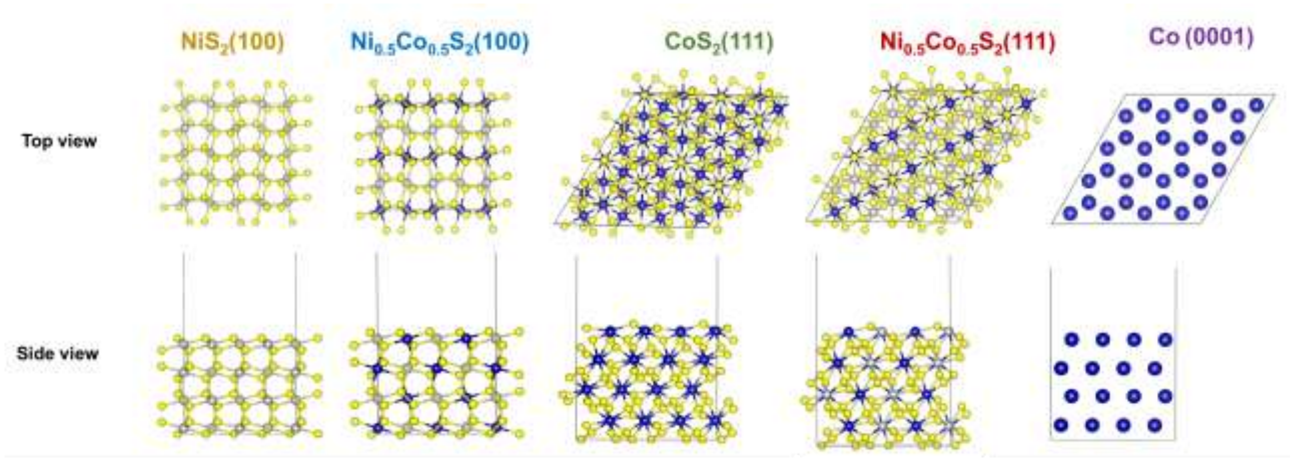

Figure S12. Top and side views of various transition metal pyrite surfaces. The grey, blue and yellow spheres represent Ni, Co, and S atoms.

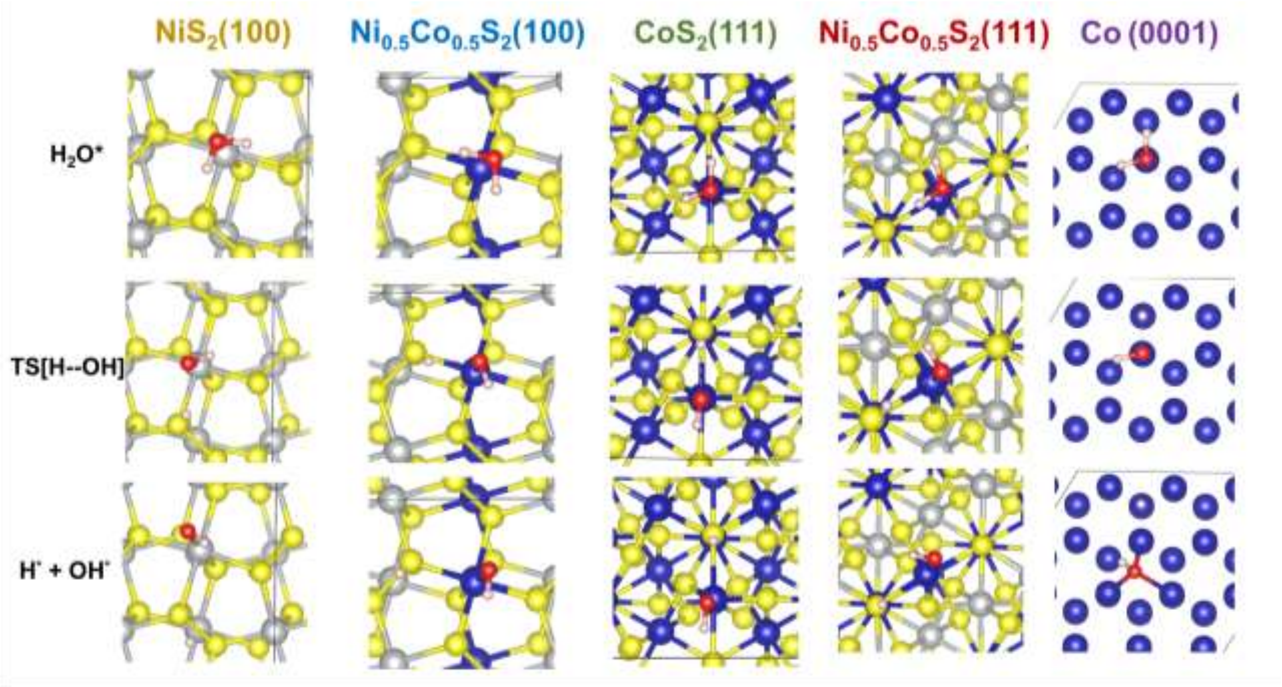

Figure S13. Optimized structures of initial, transition, and final states for breaking the H-OH bond in the Volmer step on various surface.

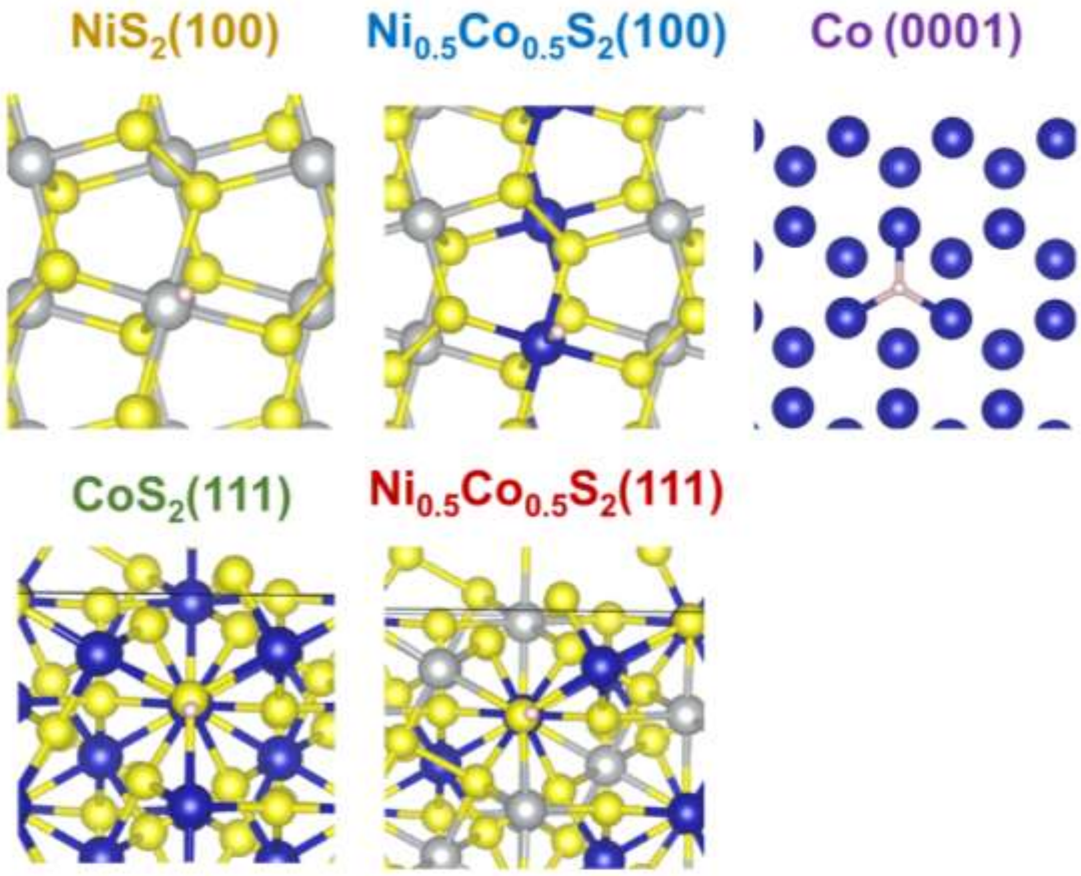

Figure S14. Optimized structures of H adsorption on various surfaces.
